# BTP2 restricts Tulane virus and human norovirus replication independent of store-operated calcium entry

**DOI:** 10.1128/jvi.00444-25

**Published:** 2025-05-29

**Authors:** Francesca J. Scribano, J. Thomas Gebert, Kristen A. Engevik, Nicole M. Hayes, Jorge Villanueva, Son Pham, Soni Kaundal, Janam J. Dave, B. V. Venkataram Prasad, Mary K. Estes, Sasirekha Ramani, Joseph M. Hyser

**Affiliations:** 1 Department of Molecular Virology and Microbiology, Baylor College of Medicine 189531, Houston, Texas, USA; 2 Alkek Center for Metagenomics & Microbiome Research, Baylor College of Medicine 661296, Houston, Texas, USA; 3 Verna and Marrs McLean Department of Biochemistry and Molecular Pharmacology, Baylor College of Medicine 3989, Houston, Texas, USA; University of Kentucky College of Medicine, Lexington, Kentucky, USA

**Keywords:** Tulane virus, human norovirus, antiviral, BTP2

## Abstract

Human norovirus is the leading cause of viral gastroenteritis across all age groups. While there is a need for human norovirus antivirals, therapeutic development has been hindered by a lack of cell culture systems and animal models of infection. Surrogate viruses, such as Tulane virus (TV), have provided tractable systems to screen potential antiviral compounds. Our previous work demonstrated that TV encodes a viral ion channel, which dysregulates cytosolic calcium signaling. We set out to investigate whether host pathways triggered by viral ion channel activity, including store-operated calcium entry (SOCE), play a role in virus replication. Using pharmacologic inhibitors and genetically engineered cell lines, we establish that the SOCE inhibitor, BTP2, reduces TV replication in an SOCE-independent manner. We observed a significant reduction in TV replication, protein expression, and RNA synthesis in cells with both pre- and post-infection BTP2 treatment. By serial passage and plaque isolation, we demonstrate that TV quasi-species have mixed susceptibility and resistance to BTP2. Sequence comparison of the quasi-species revealed that amino acid changes in the structural proteins were associated with drug resistance. We utilized reverse genetics to generate TV with the resistance-associated VP1 and VP2 amino acid changes and found that amino acid changes in both proteins conferred BTP2 resistance. Together, this supports that TV structural proteins are the targets of BTP2. Finally, using human intestinal organoids, we demonstrate that BTP2 significantly reduces human norovirus replication.

**IMPORTANCE:**

Our work identifies BTP2 as a potential human norovirus antiviral pharmacophore and highlights the utility of targeting calicivirus structural proteins to restrict viral replication. Furthermore, we establish a system whereby Tulane virus (TV) can be used to screen novel antiviral candidates and establish their mechanism of action. Together, this will facilitate rapid preclinical validation of other novel human norovirus therapeutics.

## INTRODUCTION

Human norovirus (HuNoV) is the leading cause of non-bacterial gastroenteritis across all age groups (1, 2). While HuNoV infection is acute and self-limiting in immunocompetent hosts, severe, chronic infections occur in vulnerable populations, including children, the elderly, and immunocompromised patients. In the United States, HuNoV infection accounts for 465,000 emergency department visits and 109,000 hospitalizations each year (3, 4). Globally, HuNoV causes about 212,000 annual deaths (5). To date, there are no approved prophylactic or therapeutic treatments that inhibit HuNoV replication or HuNoV-associated disease.

The development of HuNoV vaccines or antiviral therapies has been complicated by a limited understanding of the mechanisms of viral replication and pathogenesis. This is in large part due to historical challenges in propagating HuNoV in traditional cell culture systems and animal models. Currently, models for antiviral screening include surrogate virus systems such as Tulane virus (TV) or murine norovirus (MNV), the HuNoV replicon, and bona fide HuNoV replication in human intestinal organoids (HIOs) or zebrafish larvae. The recent finding that HuNoV can infect HIOs and zebrafish larvae has revolutionized the norovirus field (6–12). However, while these systems productively replicate multiple GI and GII HuNoV genogroups (6, 7), they can be costly for high-throughput antiviral screens or technically challenging to work with. Additionally, HIOs do not currently replicate HuNoVs with yields high enough to support continuous passage or production of virus stocks, so samples for virus inoculation are limited to patient stool. Therefore, utilizing surrogate viruses, including TV or MNV, can be useful to better understand mechanisms of replication, which can then be validated in more directed organoid studies with HuNoV, as has been done with virus disinfectant and inactivation studies (13–15).

TV, the prototype strain of the recovirus genus, was isolated from a rhesus macaque in 2008. Challenge studies suggest that TV-infected macaques present with a gastroenteritis-like disease, characterized by fever, diarrhea, duodenal inflammation, and virus shedding in the stool (16). As new strains of recoviruses are identified, there is mounting evidence that some strains can be detected in human stool (17), neutralized by cross-reactive antibodies in human sera (18), and cultivated in continuous *in vitro* human cell lines (19). Whether recoviruses contribute to the global burden of gastroenteritis in humans remains undetermined. However, recoviruses share many similarities with HuNoV and are a useful tool to investigate important aspects of norovirus biology. TV is a favorable surrogate model for HuNoV in that both viruses have similar genome organizations (20), bind to histo-blood group antigens (21), and cause acute diarrheal symptoms in permissive hosts (16). Additionally, unlike HuNoV, TV can productively replicate in continuous monkey kidney cell lines, such as rhesus LLC-MK2 or vervet MA104 cells.

The utility of TV in the discovery of novel aspects of calicivirus infection is underscored by the recent finding that the non-structural protein NS1-2 functions as an endoplasmic reticulum (ER)-localized viral ion channel (viroporin) (22, 23). TV NS1-2 viroporin activity dysregulates cytosolic calcium signaling within infected cells, and transfection of HuNoV NS1-2 causes a similar pattern of calcium dysregulation (22). Importantly, both ER and cytosolic calcium are important for TV replication, and we speculate that the same may be true for HuNoV (22). While analogous viroporins, such as rotavirus NSP4, have been shown to promote virus replication (24–27), how dysregulation of intracellular calcium signaling aids in calicivirus replication remains unknown. In addition to directly affecting virus replication, viroporin activity engages host cell processes involved in maintaining calcium homeostasis, which independently may have important consequences for the virus life cycle. One such pathway is store-operated calcium entry (SOCE), which is triggered upon ER calcium store depletion. Two proteins, STIM and Orai, together are both necessary and sufficient for capacitive calcium entry through the SOCE pathway (28–32). The STIM proteins (STIM1 and STIM2) function as ER calcium sensors, whereas Orai channels, comprised of homo- or hetero-oligomers of Orai1, Orai2, and Orai3, allow for calcium influx. When ER calcium concentrations fall, calcium ions dissociate from the EF hand domains of STIM, with STIM1 serving as the main calcium sensor isoform in most cells (29, 33). This dissociation triggers STIM1 oligomerization and facilitates translocation to ER-plasma membrane junctions (34). At these sites, STIM1 triggers calcium influx through Orai (35). Following a local increase in cytosolic calcium, these ions are pumped back into the ER through the sarcoplasmic/endoplasmic reticulum calcium ATPase (SERCA). Previous studies have demonstrated that ER calcium release by rotavirus NSP4 activates SOCE and that inhibition of Orai or knockdown of STIM attenuates rotavirus replication (24, 26). Given the importance of this pathway in enteric virus replication and the likelihood of ER calcium release by calicivirus NS1-2 viroporin activity, we became interested in the role of SOCE in TV and HuNoV infection. Here we identify that the Orai channel inhibitor, BTP2, has antiviral activity against TV and two strains of HuNoV, but demonstrate that this effect is SOCE-independent. Importantly, we also demonstrate that, as the likely targets of BTP2, the calicivirus structural proteins may be attractive targets for further antiviral development. Together, this work underscores the utility of surrogate virus systems in the discovery and evaluation of novel candidates for the development of HuNoV therapeutics.

## RESULTS

### Orai channel inhibitors block SOCE in MA104 cells but variably affect TV replication

**Fig 1:**
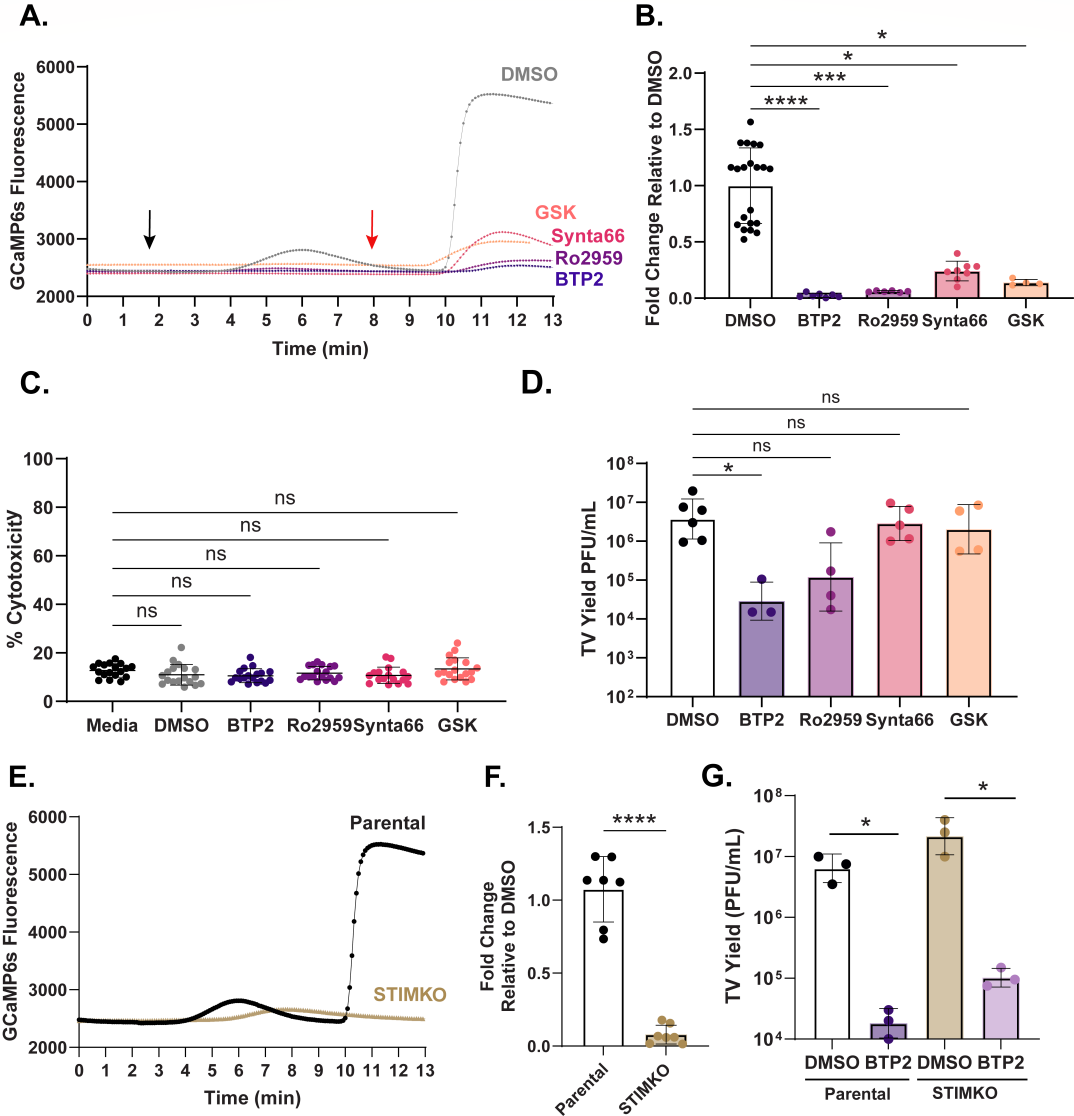
Orai channel inhibitors block SOCE in MA104 cells but variably affect TV replication. (**A**) Representative traces of GCaMP6s fluorescence following 1 µM thapsigargin treatment (black arrow) and 2 mM calcium perfusion (red arrow) in MA104G6s cells pretreated with DMSO control (gray), 5 µM Ro2959 (purple), or 10 µM BTP2 (blue), Synta66 (pink), or GSK (orange) for 20 min. (**B**) Maximum fold change in GCaMP6s fluorescence per imaging field-of-view after 2 mM calcium perfusion relative to DMSO control. All experiments were performed with a minimum of three biological repeats. (**C**) Lactate dehydrogenase (LDH)-based cytotoxicity in MA104G6s cells after 24 h treatment with media alone (black), DMSO (gray), 10 µM BTP2, Synta66, GSK, or 5 µM Ro2959. All experiments were performed with a minimum of three biological repeats of six technical replicates. (**D**) TV yield in PFU/mL 24 hours post-infection (hpi) at multiplicity of infection (MOI) 1 with BTP2, Synta66, GSK (10 µM), and Ro2959 (5 µM). All compounds were added 1 hpi. Data are shown as an average of technical duplicates across at least three biological replicates. (**E**) Representative traces of GCaMP6s fluorescence following thapsigargin treatment and 2 mM calcium perfusion in parental MA104G6s cells (black) or STIM1 KO cells (gold). (**F**) Maximum fold change in GCaMP6s fluorescence per imaging field-of-view after 2 mM calcium perfusion relative to parental cells. Data are shown as a minimum of two technical replicates of at least three biological repeats. (**G**) TV yields 24 hpi at MOI 1 in MA104G6s parental and STIM1 KO cells treated with DMSO control or 10 µM BTP2 1 hpi. Data are shown as an average of technical duplicates across at least three biological replicates. For all experiments, normality was assessed by the Shapiro-Wilk test. For SOCE and LDH experiments, ****P < 0.0001 by Kruskal-Wallis with Dunn’s multiple comparisons test after removal of outliers (ROUT Q = 1%). TV yield assay data are plotted as geometric mean ± SD. *P < 0.1 by Kruskal-Wallis with Dunn’s multiple comparisons test or unpaired T test.

We previously demonstrated that TV NS1-2 localizes to the ER and disrupts intracellular calcium signaling during infection in a manner similar to rotavirus NSP4, the prototype calcium-conducting viroporin (22). Additionally, NSP4 triggers SOCE, and blocking this pathway *in vitro* significantly inhibits rotavirus replication (24). Thus, we sought to determine the effect of several SOCE inhibitors on TV replication. We first confirmed that four well-characterized Orai channel blockers, BTP2, Ro2959, GSK-7975A (GSK), and Synta66, impair SOCE in MA104 cells by measuring cytosolic calcium concentrations during ER store depletion and calcium reintroduction (36). In this assay, we measured relative cytosolic calcium by tracking the fluorescence of the genetically encoded calcium indicator, GCaMP6s. ER calcium stores were first depleted using thapsigargin to inhibit the SERCA pump in the presence of calcium-free medium (black arrow, Fig. 1A). This enabled strong activation (i.e., opening) of SOCE channels in the plasma membrane without calcium influx into the cytoplasm. Calcium-containing media was then reintroduced, resulting in calcium entering the cell through the activated SOCE channels (red arrow, Fig. 1A). By quantifying the maximum change in GCaMP6s fluorescence due to calcium influx, we found that all the compounds tested significantly reduced SOCE compared to the dimethyl sulfoxide (DMSO) control (Fig. 1B). Additionally, there were no cytotoxic effects of these inhibitors at the tested concentrations (Fig. 1C). We next wanted to determine whether these inhibitors would impact TV replication. Although BTP2, Ro2959, GSK, and Synta66 all inhibited SOCE in MA104 cells, only BTP2 significantly reduced TV yield (Fig. 1D). Given these discordant results, we next tested whether BTP2 inhibition of TV replication was independent of SOCE activity. Using CRISPR-Cas9, we developed a line of MA104 cells with a genetic knockout of STIM1, a protein required for SOCE activation (28, 29, 37). This line showed loss of STIM1 protein expression by immunoblot (Fig. S1) and almost complete abrogation of SOCE-mediated calcium influx upon ER calcium depletion (Fig. 1E and F). Together, these data validated the STIM1 knockout and showed that there were no compensatory mechanisms for SOCE in the absence of STIM1 in these cells. Despite the lack of SOCE, the STIM1 knockout did *not* affect TV replication, and BTP2 maintained its ability to significantly reduce TV yield (Fig. 1G). This indicates that BTP2 blocks TV replication in an SOCE-independent manner. Given this, we sought to characterize BTP2-induced virus inhibition and to determine the stages of viral replication that were most significantly affected by compound treatment.

### BTP2 inhibits both early- and late-stage TV replication

**Fig 2:**
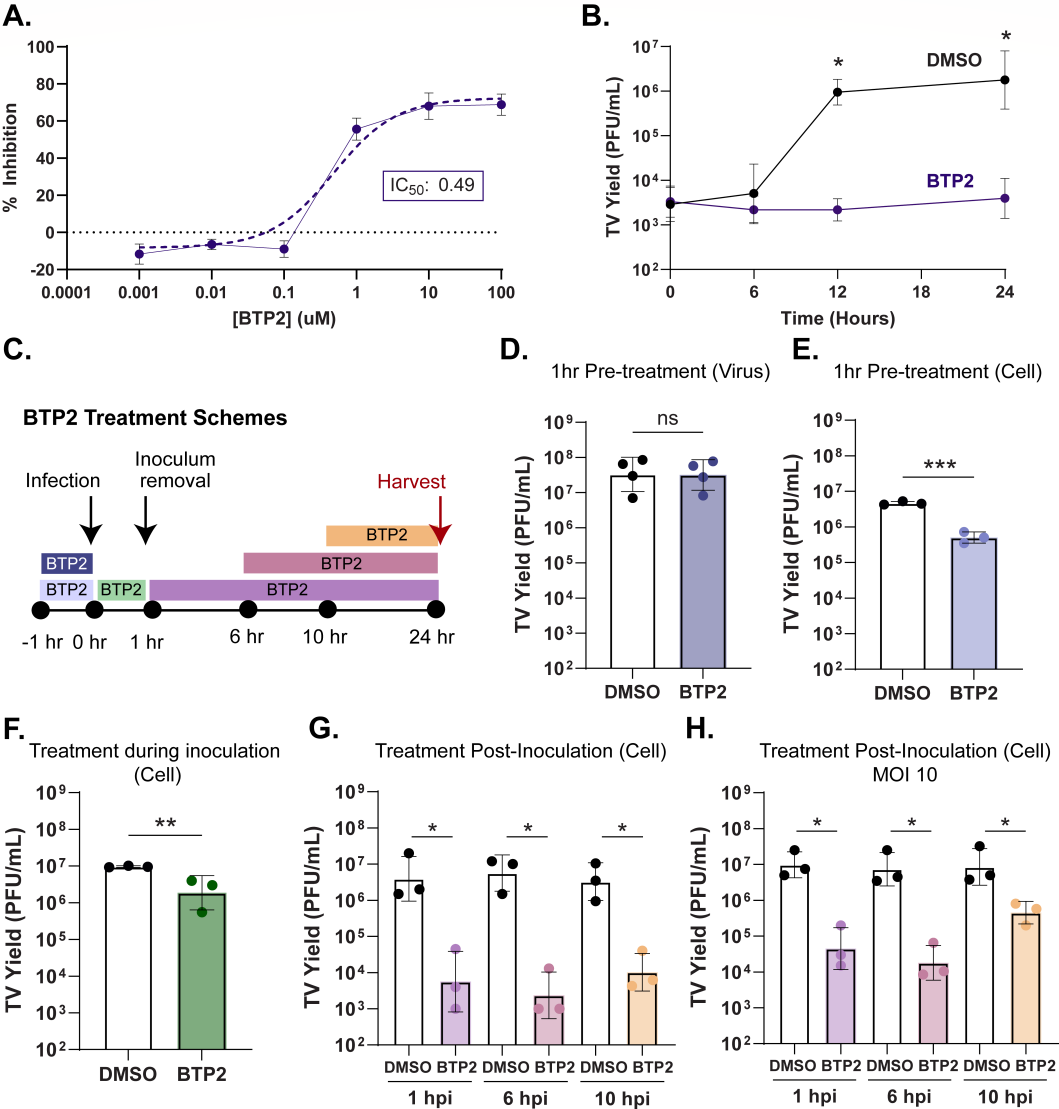
BTP2 inhibits both early- and late-stage TV replication. (**A**) Percent inhibition of monolayer clearance in crystal violet-based assay in TV-infected monolayers (MOI 10) treated with 0.001, 0.01, 0.1, 1, 10, or 100 µM BTP2. The dose curve was fit with a non-linear regression (dotted line) to estimate the inhibitory concentration 50 (IC_50_). (**B**) Time course assay measuring TV yield in PFU/mL at 1, 6, 12, and 24 hpi with DMSO (black) or BTP2 (purple) treatment at 1 hpi with an MOI of 1 infection. (**C**) Schematic of BTP2 time-of-treatment. A total of 10 µM BTP2 stock was incubated with virus or cell monolayers for 1 h prior to infection, or with cell monolayers during the 1 h inoculum incubation, or 1, 6, or 10 h post-inoculation. All infections were harvested at 24 hpi. (D–G) Plaque assay titrations of TV yield following MOI 1 infection and the corresponding pre-treatment scheme of TV (**D**) or pre- and post-treatment schemes of cell monolayers (E–G). (**H**) Plaque assay titration of TV yield after MOI 10 infection and treatment with BTP2 at 1, 6, or 10 hpi. All data are shown as an average of technical duplicates across at least three biological replicates. For all experiments, normality was assessed by the Shapiro-Wilk test. TV yield assay data are plotted as geometric mean ± SD. **P < 0.01 by Kruskal-Wallis with Dunn’s multiple comparisons test or unpaired T test.

**Fig 3:**
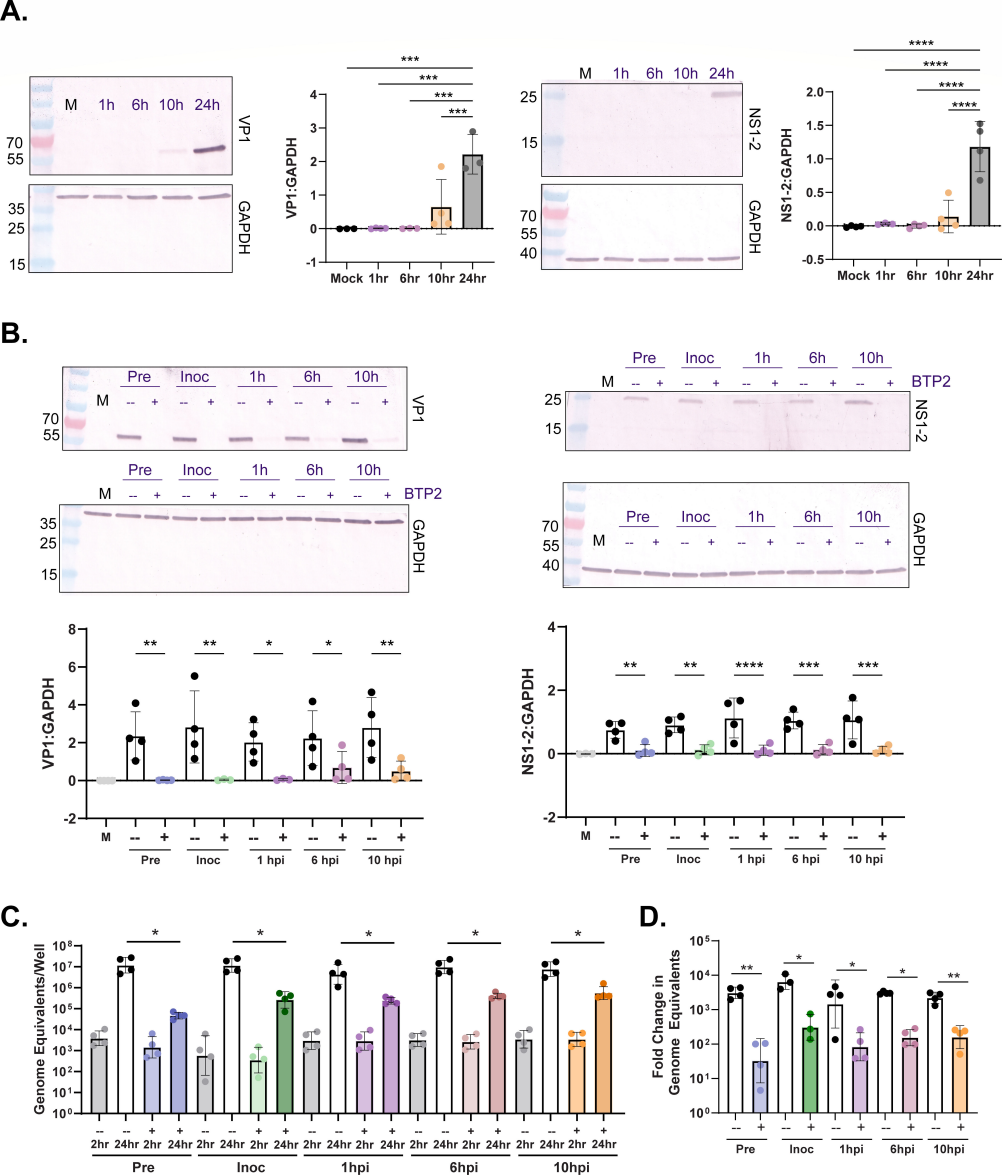
Early and late stage BTP2 addition reduces TV protein and RNA synthesis. (**A**) TV VP1 or NS1-2 protein expression detected by western blot at 1, 6, 10, or 24 hpi. (**B**) TV VP1 and NS1-2 expression at 24 h following DMSO (−) or 10 µM BTP2 (+) addition 1 h before infection (pre), during viral inoculation (inoc), or 1, 6, or 10 hpi at an MOI of 1. Glyceraldehyde 3-phosphate dehydrogenase (GAPDH) served as the loading control. For quantitation, band intensity was plotted relative to GAPDH. Each point represents an independent biological replicate. (**C**) TV genome equivalents were determined through quantitative reverse transcription PCR (RT-qPCR) of infected cells treated with DMSO (−) or BTP2 (+) at the indicated time points. RNA was harvested at 2 and 24 hpi, and genome equivalents were determined based on a standard curve from *in vitro* transcribed TV RNA. (**D**) Fold change in TV genome equivalents between 2 and 24 h with DMSO (−) or BTP2 (+) treatment at the indicated time points. Data are shown from four biological replicates, and the RT-qPCR data represent an average of technical duplicates for each biological replicate. ****P < 0.0001 by one-way analysis of variance (ANOVA) with Dunnett’s or Sidak’s multiple comparisons test.

To better assess the effect of BTP2 on TV infection in MA104 cells, we first performed a dose range study and determined the 50% inhibitory concentration (IC_50_), which was achieved with a BTP2 concentration of ~0.49 µM (Fig. 2A). Given that there was no additive inhibitory effect of BTP2 above 10 µM (~20× IC_50_), we used this concentration for our characterization studies, unless otherwise specified. To better characterize TV replication kinetics and BTP2 antiviral activity, we performed a time-course TV yield assay, adding BTP2 at 1 hour post-infection (hpi) and measuring virus yield at 0, 6, 12, and 24 hpi. BTP2 significantly reduced TV yield at 12 and 24 hpi (Fig. 2B). To better define the stages of virus replication most significantly inhibited by compound treatment, we treated the virus or the cell monolayers with 10 µM BTP2 at specified time points before, during, or after infection (Fig. 2C). Direct incubation of TV with BTP2 for 1 h did not affect the ability of the virus to infect and replicate compared to the DMSO control (Fig. 2D), indicating that BTP2 did not neutralize virus particles. By contrast, incubating *cell monolayers* with BTP2 for 1 h before or during TV inoculation significantly reduced replication (Fig. 2E and F). Likewise, adding BTP2 to the cell monolayers at 1 hpi and as late as 6 and 10 hpi significantly reduced virus yield (Fig. 2G). Since these yield assays were performed at MOI 1, we further tested whether the effect of post-infection treatment might be due to inhibition of the early stages of secondary rounds of replication. Thus, we repeated the time of addition yield studies with an MOI of 10 infection. These results mirrored our MOI 1 studies, in that BTP2 addition at 1, 6, and 10 hpi significantly reduced replication (Fig. 2H), suggesting that the inhibitory effect occurs throughout the initial round of replication. This supports a model in which BTP2 treatment reduces replication kinetics in addition to inhibition of early events in the replication cycle. To determine if BTP2 impairs viral protein synthesis, we repeated the above yield assays and quantified TV VP1 and NS1-2 expression by western blot. Detectable TV VP1 and NS1-2 expression was seen by 10 hpi and continued to increase through 24 hpi (Fig. 3A). Like the TV yield studies, BTP2 treatment prior to, during, or at 1, 6, or 10 h post-inoculation reduced TV protein expression (Fig. 3B). To determine the effect of BTP2 on genome replication, we measured TV genome equivalents at 2 and 24 hpi with the same BTP2 treatment schemes. Paralleling our yield and protein expression data, pre- and post-infection BTP2 treatment significantly reduced levels of TV RNA at 24 hpi and genome replication between 2 and 24 hpi (Fig. 3C and D). Together, these data suggest that BTP2 inhibits the viral life cycle at a point prior to translation and genome replication.

### TV overcomes BTP2 susceptibility with passage through the emergence of a resistant variant

**Fig 4:**
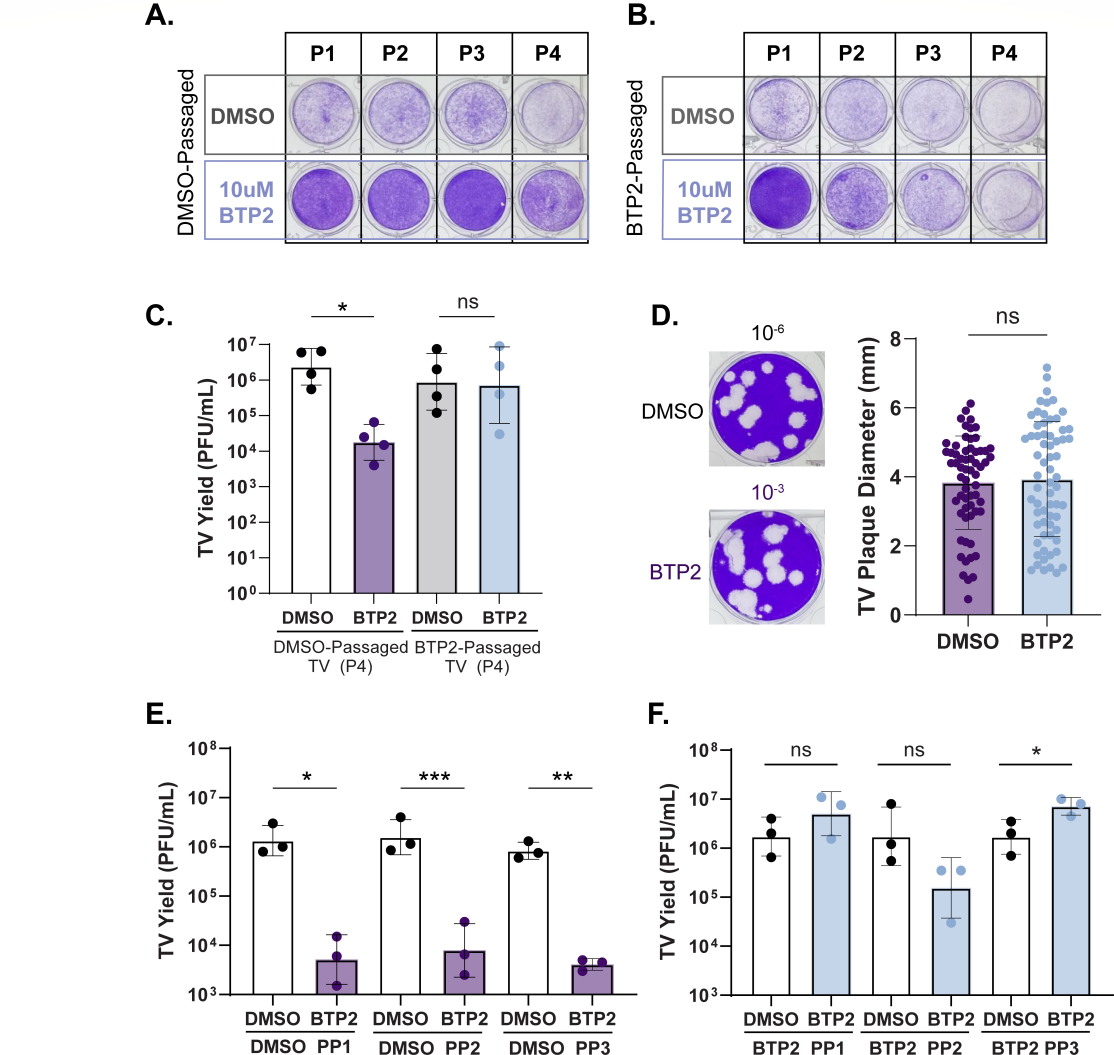
TV overcomes BTP2 susceptibility with passage through the emergence of resistant quasi-species. (**A**) Representative images of crystal-violet-stained MA104 cell monolayers after infection with DMSO-passaged TV (A, passage [P] 1–4) or BTP2-passaged TV (B, passage [P] 1–4) in the presence of DMSO or 10 µM BTP2. (**C**) TV yield in PFU/mL from passage 4 DMSO or BTP2-passaged virus, challenged with DMSO (white, gray) or 10 µM BTP2 (purple, light blue). Data are shown as an average of technical duplicates across at least three biological replicates. (**D**) Representative images of crystal violet-stained plaques following DMSO or 5 µM BTP2 addition to the plaque assay overlay (left panel) and quantitation of plaque diameter (millimeters) across three independent infections (right panel, *n* > 62 plaques). (E and F) TV yield in PFU/mL from DMSO (**E**) or BTP2 (**F**) solid overlay plaque picks, challenged with DMSO (white) or 10 µM BTP2 (purple). Data are shown from at least three biological replicates. For all experiments, normality was assessed by the Shapiro-Wilk test. TV yield assay data are plotted as geometric mean ± SD. **P < 0.01 by Mann-Whitney or unpaired T test. Plaque diameter data were analyzed by the Mann-Whitney U test.

The time-of-addition experiments suggested that BTP2 treatment inhibits pre-transcriptional and/or translational phases of the viral life cycle; however, they did not allow for the determination of whether specific viral proteins are inhibited. To determine if there was a viral target of BTP2, we serially passaged TV in the presence of BTP2 to rescue a BTP2-resistant mutant. We used crystal violet to stain infected cell monolayers and observed that DMSO-passaged virus, at every passage, caused complete cytopathic effect (CPE) when treated with the DMSO vehicle control. In contrast, in the presence of BTP2, DMSO-passaged virus caused little CPE, suggesting that BTP2 protected cells from TV-induced cell death (Fig. 4A). Conversely, BTP2-passaged TV caused CPE when treated with DMSO but became increasingly resistant to BTP2-mediated monolayer protection with each passage (Fig. 4B). We confirmed by virus yield assay, using passage 4 virus, that the DMSO-passaged virus maintained its BTP2 susceptibility, while the BTP2-passaged virus was BTP2 resistant (Fig. 4C). We considered two possibilities for the isolation of BTP2-resistant TV: it may have arisen either by *de novo* mutation or by selection of naturally resistant quasi-species in our virus stock. Since BTP2 resistance emerged after only four passages in culture, we suspected the latter. To test this, we performed plaque assays with *non-drug passaged* virus stock, adding BTP2 or DMSO control to the overlay. In the presence of BTP2, TV plaques were still observed at dilutions of 10^−3^ or lower (Fig. 4D), but in the presence of the DMSO vehicle, plaques were observed out to the 10^−6^ dilution. Additionally, the plaques that were formed from both treatments showed no difference in plaque size (Fig. 4D). Together, these data indicate that the virus that initiated the plaques in each respective treatment had no defect in multi-round viral spread. By extension, BTP2-resistant virus was present in our non-passaged stocks, although at far lower concentrations than the BTP2-susceptible virus. To confirm the presence of BTP2-resistant virus in our non-passaged stocks, we isolated virus from the recovered plaques and found that the plaque picks from wells with BTP2-containing overlay were BTP2 resistant, while plaque picks from wells with DMSO-containing overlay were BTP2 susceptible (Fig. 4E and F). These results confirmed that our starting viral stock contained a mixture of BTP2-susceptible and resistant TV quasi-species.

### Amino acid changes in TV VP1 and VP2 are associated with BTP2 resistance

**Fig 5:**
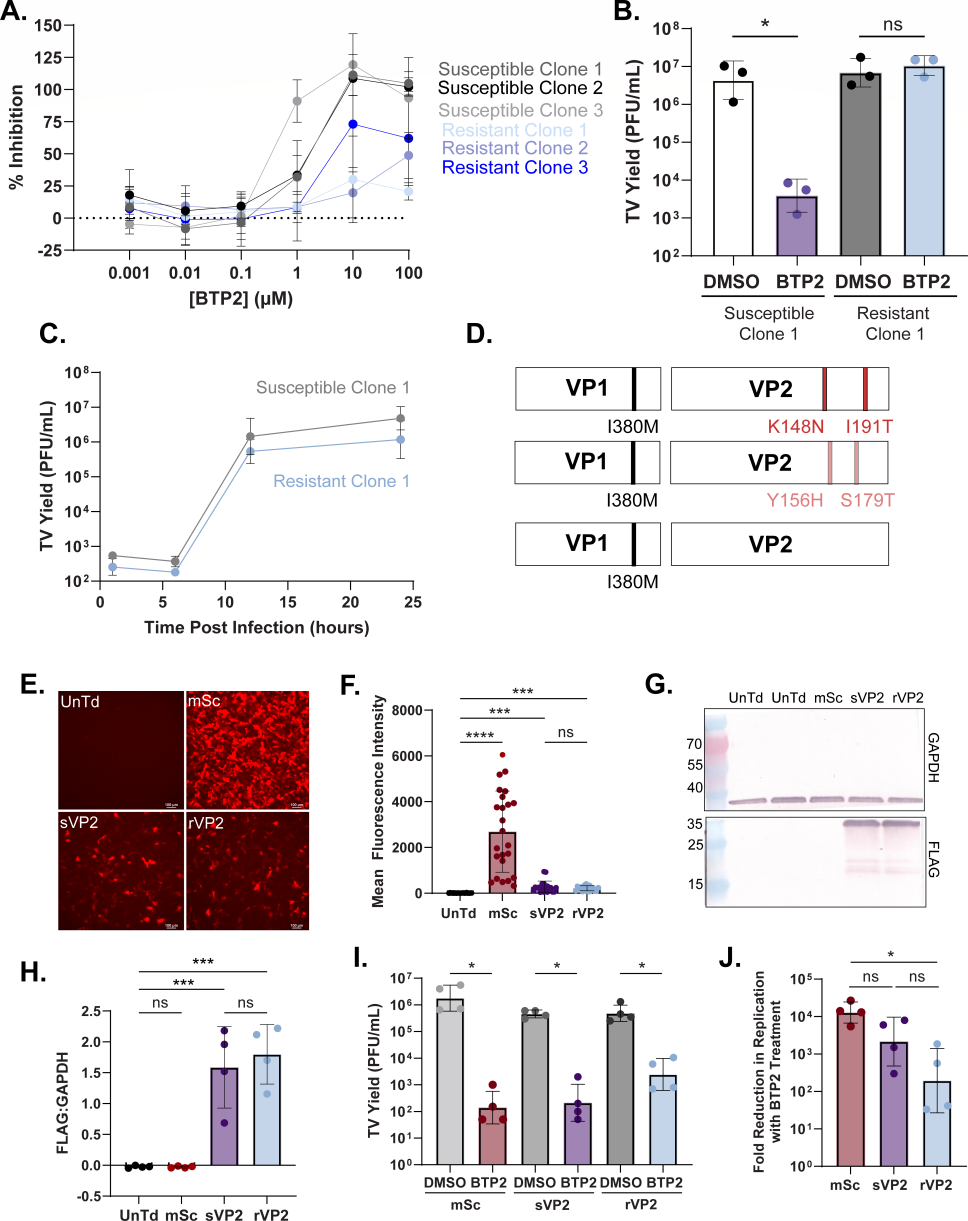
Amino acid changes in TV VP1 and VP2 are associated with BTP2 resistance. (**A**) Percent inhibition of monolayer clearance in crystal violet-based assay with three serial plaque-isolated susceptible viruses (gray) and three serial isolated resistant viruses (blue) (MOI 3) treated with a dose range of BTP2. (**B**) TV yield assay with one serial plaque-isolated susceptible virus (clone 1) and one serially isolated resistant virus (clone 1) challenged with DMSO or 10 µM BTP2. (**C**) Time course assay measuring yield at 1, 6, 12, and 24 hpi with one serial plaque-isolated susceptible virus (clone 1, gray) and one serially isolated resistant virus (clone 1, blue). (**D**) Schematic of amino acid differences identified between BTP2-susceptible and resistant clones. (**E**) Representative images of mean fluorescence intensity of cells transduced with adeno-associated viral (AAV) vectors expressing VP2 or mScarlet alone. Images were taken at 48 hours post-transduction (hpt). Scale bar = 100 µM. (**F**) Quantitation of mean fluorescence intensity across four biological replicates. (**G**) Western blot demonstrating expression of FLAG-tagged susceptible or resistant VP2 following AAV transduction. Cell lysates were harvested at 48 hpt. (**H**) Quantitation of western blot band intensity plotted relative to GAPDH. Each point represents an independent biological replicate. (**I**) Plaque assay titration of MA104 cells transduced with AAVs expressing mScarlet or susceptible or resistant VP2, infected with BTP2-susceptible TV (susceptible clone 1) at an MOI of 1, and treated with DMSO control or BTP2. Infections were performed 48 hpt, and compound treatment was done 1 hpi. (**J**) Fold change in virus yield between DMSO and BTP2 treatment conditions from panel I. For all AAV experiments, data were collected across four biological replicates. Normality was assessed by the Shapiro-Wilk test. TV yield assay data are plotted as geometric mean ± SD, *P < 0.05 by unpaired T test. Mean fluorescence intensity data is compared using Kruskal-Wallis with Dunn’s multiple comparisons test. For the fold change and western blot quantitation, *P < 0.05 by one-way ANOVA with Sidak’s multiple comparisons test.

The BTP2 plaque isolation experiments allowed us to distinguish multiple BTP2-susceptible and resistant TV variants, and we sought to determine whether they contained resistance-associated amino acid differences. To isolate clonal populations of susceptible and resistant virus, we performed three rounds of serial plaque isolation in the absence or presence of BTP2. We utilized virus inhibition assays to determine the degree of susceptibility of each of the clones to BTP2 treatment (Fig. 5A). We noted that one of the BTP2 isolated clones, resistant clone 3, had an intermediate susceptibility phenotype (Fig. 5A). We next performed yield assays with one representative susceptible and one representative resistant clone to verify that the BTP2 serial plaque isolation yielded BTP2-resistant virus, while the DMSO serial plaque isolation yielded BTP2-susceptible virus (Fig. 5B). To determine if BTP2 resistance conferred a fitness cost or benefit, we performed growth curves using the same representative resistant and susceptible isolate. We found that BTP2 susceptibility does not affect the ability of these viruses to grow in cell culture in the absence of treatment (Fig. 5C). Given the distinct effects of BTP2 treatment on these isolated viral variants, we utilized Sanger sequencing to identify sequence differences associated with resistance. In total, we detected one amino acid difference in VP1 (I380M), which was universally represented in all of the BTP2-resistant clones and a cluster of non-uniform amino acid differences at the C-terminus of VP2, which were present in two of the resistant clones (clone 1: K148N, I191T; clone 2: Y156H, S179T) (Fig. 5D; Fig. S2 and S3). No conserved amino acid changes were found in the ORF1 non-structural proteins in any of the clones. This finding is consistent with cell-free functional assays that showed that BTP2 treatment did not affect TV or HuNoV protease activity (Fig. S4A and B) or HuNoV polymerase activity (Fig. S4C). While there is no available structure of TV VP2, mapping of the I(380) residue onto the TV VP1 cryo-EM structure shows that it is positioned at the VP1 dimer interface (Fig. S5B).

Given that the amino acid differences in VP2 were non-uniform, we sought to validate whether VP2 played a role in mediating BTP2 resistance. To do this, we performed co-transduction and infection studies and determined if overexpression of the resistant VP2 was able to confer BTP2 resistance to the plaque-isolated BTP2-susceptible virus clone. We generated adeno-associated viral (AAV) vectors for recombinant expression of VP2 from either susceptible or resistant clone 1. The VP2 from these constructs has an N-terminal 3X FLAG-tag and expressed mCherry downstream of an encephalomyocarditis virus internal ribosome entry site to aid in quantitating protein expression and transduction efficiency. We utilized an AAV vector expressing only the fluorescent protein, mScarlet, as an irrelevant protein overexpression control. We first validated AAV transduction efficiency by measuring mScarlet or mCherry fluorescence and FLAG by western blot (Fig. 5E through H), which confirmed that the susceptible and resistant VP2 constructs were expressed to similar levels. Next, we determined the yield of a BTP2-susceptible clone of TV following infection of mScarlet or VP2-transduced monolayers treated with DMSO or BTP2. We found that in all cases, BTP2 treatment significantly reduced viral yield (Fig. 5I). We next analyzed the fold reduction in TV replication between DMSO and BTP2 treatment for each transduction condition. We found that the degree of BTP2 inhibition was statistically lower in cells expressing the resistant VP2 than those expressing mScarlet, but it was not statistically different between monolayers expressing mScarlet and the susceptible VP2 or between susceptible VP2 and resistant VP2 expression (Fig. 5J). Thus, while the expression of the resistant VP2 did have a modest effect on the BTP2 sensitivity of TV, ultimately it does not confer complete resistance to BTP2 treatment.

### TV structural proteins mediate BTP2 susceptibility

**Fig 6:**
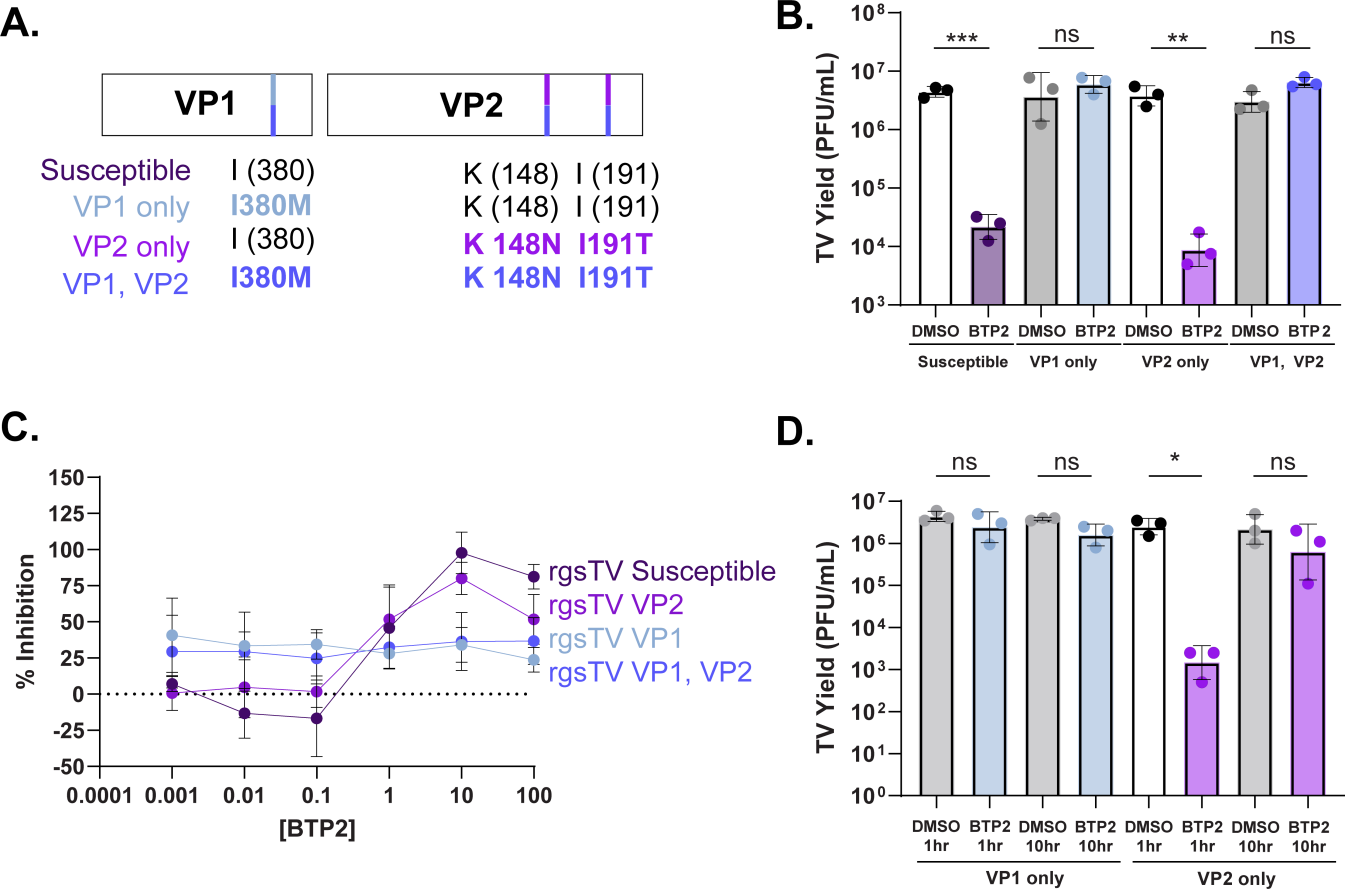
TV structural proteins mediate BTP2 susceptibility. (**A**) Schematic of susceptible or resistance-associated amino acids in the four recombinant Tulane virus variants. (**B**) Recombinant TV yield at 24 hpi following an MOI 1 infection with DMSO or 10 µM BTP2 treatment at 1 hpi. (**C**) Percent inhibition of monolayer clearance with recombinant TV (MOI 3) infection and treatment with BTP2 at 1 hpi at the indicated dose range. (**D**) Recombinant TV yield at 24 hpi following an MOI 1 infection with DMSO or 10 µM BTP2 treatment at 1 or 10 hpi. For yield experiments, normality was assessed by the Shapiro-Wilk test, and data are plotted as geometric mean ± SD. ***P < 0.001 by unpaired T test.

To further assess the role of the structural proteins in BTP2 resistance, we utilized reverse genetics to rescue TV with the resistance-associated amino acid changes in VP1 only, VP2 only, or both VP1 and VP2. The susceptible and resistance-associated sequences were generated from susceptible clone 1 and resistant clone 1 (Fig. 6A; Fig. S5A and S6A). As expected, the rescued TV with BTP2 susceptibility-associated VP1 and VP2 showed significantly reduced yield with BTP2 treatment at 1 hpi (Fig. 6B). Recombinant TV with the VP2-only resistance-associated amino acid changes also demonstrated susceptibility to early BTP2 treatment. However, the viruses with the VP1 only and dual VP1, VP2 resistance-associated amino acid changes were BTP2 resistant (Fig. 6B). There were no significant differences in susceptibility to 1 hpi BTP2 treatment across a range of doses between the VP1 only and combination VP1, VP2 viruses or the susceptible and VP2 only viruses (Fig. 6C). Our time-of-addition data suggested that BTP2 may be functioning through independent mechanisms of inhibition during early and late stages of infection. Thus, we next wanted to evaluate the susceptibility of our different recombinant TV variants when BTP2 was added at different time points post-infection. Surprisingly, while the VP1 only virus maintained BTP2 resistance when the compound was added both early (1 hpi) and late (10 hpi) in infection, the VP2 only virus showed BTP2 sensitivity upon early addition, but resistance with late addition (Fig. 6D). Together, these data show that VP1 and VP2 changes are both capable of conferring BTP2 resistance, although through potentially distinct mechanisms of action.

### BTP2 inhibits human norovirus replication

**Fig 7:**
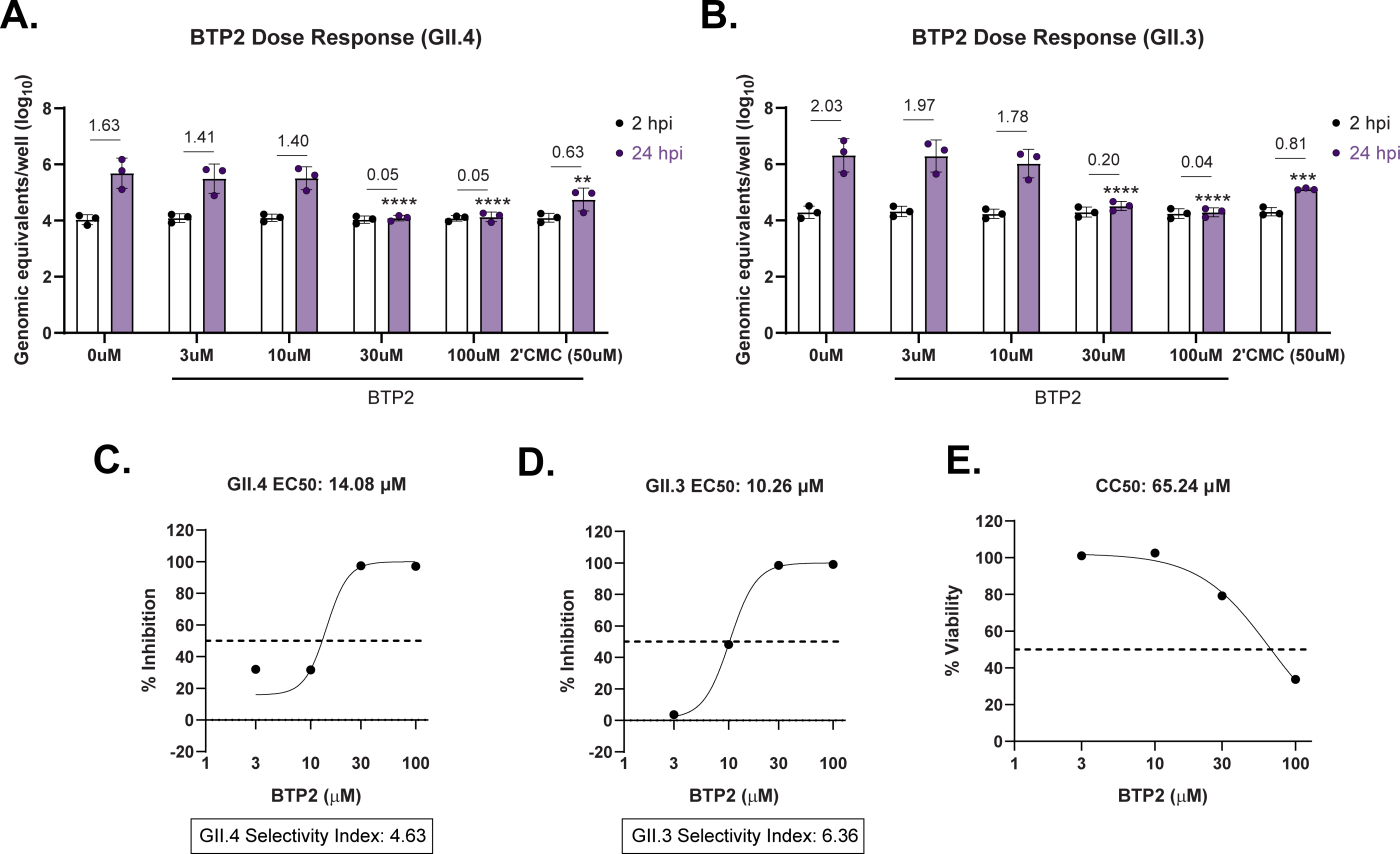
BTP2 inhibits HuNoV replication. (A) Quantitation of HuNoV genome equivalents in jHIOs inoculated with 100 TCID50s of GII.4 virus at 2 and 24 hpi after treatment with a dose range of BTP2 or 50 µM 2-CMC. Both compounds were incubated with enteroid monolayers for 1 h before infection and maintained in the maintenance media until harvest (24 h). (**B**) Quantitation of HuNoV genome equivalents in jHIOs inoculated with 100 TCID50s of GII.3 (P21) virus at 2 and 24 hpi. jHIOs were treated with BTP2 or 2-CMC as described above at the indicated concentrations. (C and D) Percent inhibition of GII.4 (**C**) or GII.3 (**D**) replication across the BTP2 dose range for three independent experiments. EC_50_ and CC_50_ values were calculated using non-linear regression, and the selective indices were calculated by dividing the CC_50_ by the EC_50_. (**E**) Percent viability as measured by LDH assay over a dose range of BTP2. Data are pooled from five independent experiments. For HuNoV replication data, ****P < 0.0001 by two-way ANOVA with Dunnett’s multiple comparisons test.

Given that BTP2 inhibited TV replication, we next assessed BTP2 activity against HuNoV using our antiviral pipeline for HIOs (38). We first evaluated GII.4 Sydney (P16) HuNoV replication in jejunum-derived human intestinal organoids (jHIOs) treated with a dose range of BTP2. We found that 30 µM and 100 µM BTP2 led to a significant reduction in HuNoV replication at 24 hpi, as did the positive control, 50 µM 2′-C-methylcytidine (2-CMC) (38, 39) (Fig. 7A). While GII.4 HuNoV is the predominant circulating genotype, we wanted to evaluate the efficacy of BTP2 against other HuNoV strains. We found that 30 and 100 µM BTP2 treatment also significantly inhibited GII.3 (P21) replication in jHIOs (Fig. 7B). To evaluate the selective index of BTP2 in organoids, we plotted the percent inhibition of viral replication and the percent cytotoxicity in organoids across the BTP2 dose range. We used non-linear regression to determine the half-maximal effective concentration (EC_50_) and half-maximal cytotoxic concentration (CC_50_). By taking the ratio of these metrics, BTP2 had a selective index of 4.63 for GII.4 Sydney (P16) and a selective index of 6.36 for GII.3 (P21) HuNoV (Fig. 7C through E). These data suggest that BTP2 has weak antiviral activity against both GII.4 and GII.3 strains of HuNoV.

## DISCUSSION

There is a growing body of research that demonstrates a potential use for Orai channel blockers in infectious diseases, as they have shown *in vitro* efficacy in inhibiting virus replication (24). Viruses across a range of families encode calcium-conducting viral ion channels, which can mediate the release of calcium from the endoplasmic reticulum into the cytosol (40). In multiple contexts, this has been shown to engage the SOCE pathway. We speculated that Orai would be an attractive broad-spectrum host-directed antiviral therapeutic target. Interestingly, BTP2 was unique in its ability to block TV replication, and this was likely due to direct effects on TV structural proteins rather than on SOCE. Furthermore, BTP2 treatment significantly decreased HuNoV replication. This work identifies the calicivirus structural proteins as viable targets for antiviral development. While future work is needed to confirm that BTP2 acts directly on HuNoV structural proteins, these findings lay the foundation for the characterization of BTP2 as a novel antiviral pharmacophore.

Identifying the TV structural proteins as likely targets of BTP2 allows us to speculate on the potential mechanism by which it can inhibit viral replication. TV VP1 plays an important role in HBGA attachment factor binding (21) and capsid assembly. HuNoV VP1 similarly mediates HBGA binding (41–44) and capsid assembly (45, 46) but is also involved in viral entry (47). Less is known about the role of VP2 for both viruses, although it has been shown to stabilize HuNoV virus-like particles (48), facilitate genome encapsidation (49), and potentially regulate the activity of the RNA-dependent RNA polymerase (50). In feline calicivirus and murine norovirus, VP2 has also been shown to be essential for infectivity (51, 52). These ascribed functions of VP1 and VP2 seem consistent with a dual role for BTP2 in both the early and late stages of infection. Thus, we postulate that BTP2 may be targeting capsid stability, pioneer rounds of translation or genome replication (early), and/or capsid assembly (late) during infection. This is supported by our time-of-addition data, which indicates that treatment with BTP2 either before or after infection significantly reduced TV replication. However, we cannot rule out the possibility that the effect of pre-treatment stems from BTP2 retention within the cells even after media change, allowing BTP2 to exert its antiviral activity later during infection. A multi-modal inhibitory effect of BTP2 is also supported by our reverse genetics data, which demonstrates that there were differences in the sufficiency of VP1 or VP2 amino acid changes to confer BTP2 resistance, depending on whether BTP2 was introduced at early or late stages of infection. However, more directed studies are needed to decipher the mechanism(s) responsible for the reduction in replication observed with BTP2 treatment.

Our serial plaque isolation experiments yielded three BTP2-resistant clones with the VP1(I380M) mutation and one clone (BTP2-resistant clone 3) with no amino acid changes in VP2. Interestingly, resistant clone 3 exhibited moderate BTP2 resistance compared to the other two BTP2-resistant clones, both of which had additional mutations in VP2. Thus, we aimed to confirm the roles of both VP1 and VP2 in mediating BTP2 sensitivity by establishing an AAV overexpression system and a new plasmid-based reverse genetics system. Ectopic overexpression of the resistant VP2 was not sufficient to fully rescue the replication of clonally susceptible TV in the presence of BTP2. However, a partial rescue was seen compared to the expression of mScarlet alone. In addition, our reverse genetics studies indicate that both VP1 and VP2 resistance-associated amino acid changes are indeed resistance-conferring. The rescue of TV replication in the presence of BTP2 with VP1 only and VP2 only amino acid changes also suggests that other viral mutations or features (e.g., 5’ or 3’ UTRs) are not necessary for BTP2 resistance. Further work using TV reverse genetics to systematically test the various VP2 mutations with and without the VP1 I380M mutation will be critical to dissect the combined and individual contributions of these changes. While we find it most likely that BTP2 acts directly upon VP1 and/or VP2, there is also the possibility that BTP2 alters a host pathway in a manner that inhibits susceptible TV, but the amino acid changes in VP1 and/or VP2 in the resistant TV circumvent viral dependence on these pathways. Thus, further investigation of how BTP2 inhibits TV replication and how the amino acid changes in VP1 and VP2 confer resistance to BTP2 will provide new mechanistic insights into calicivirus replication strategies in addition to identifying new functional targets for antiviral drug development.

Given that HuNoV and TV VP1 and VP2 share only ~30% and ~27% amino acid identity, respectively, it is challenging to assess whether the mechanism of action of BTP2 is conserved across these viruses using sequencing data alone. Thus, further work is needed to verify that the HuNoV structural proteins mediate BTP2 susceptibility in the same way that we hypothesize to be true for TV. Identifying the defining features of BTP2 susceptibility in HuNoV would be particularly important, as it may allow us to predict which circulating strains of virus will be most potently inhibited. Additionally, these findings would have important implications for understanding the likelihood of, and potential barriers to, a virus acquiring *de novo* BTP2 resistance. Finally, identifying the precise mechanism of BTP2 resistance would facilitate optimized drug design to impede resistance development.

Our results are consistent with previous work, which identifies BTP2 as a known potent inhibitor of SOCE in both T cells (53, 54) and MA104 cells (26). While the mechanism of BTP2 inhibition of SOCE has yet to be fully characterized, T cell studies indicate that BTP2 acts extracellularly to inhibit Orai channel activity (53). Our time-of-addition studies are most consistent with the ability of BTP2 to permeate MA104 cells after prolonged treatment. Given that SOCE assays are done with acute treatment, it is likely that BTP2 maintains both an extracellular function in inhibiting Orai and an intracellular function in inhibiting viral replication. This work is also not the first to examine the potential effects of BTP2 on virus replication. We have previously demonstrated that BTP2 reduces rotavirus replication (26). While it is tempting to speculate that this may be due to SOCE-independent effects, STIM1 knockdown also reduces rotavirus replication (24), which we did not observe to be the case for TV. Thus, we cannot fully rule out an on-target, SOCE-mediated role for BTP2 in rotavirus infection. Other cellular off-target effects of BTP2 have also been previously identified, including inhibition of the actin reorganizing protein, drebrin (55), inhibition of transient receptor potential cation (TRPC) channels (56), and potentiation of the non-selective cation channel, TRPM4 (57). The contributions of these off-target effects to the ascribed inhibitory action of BTP2 against either SOCE or viral replication must be fully explored to understand the BTP2 antiviral mechanism.

Given both the established on- and off-target effects of BTP2, understanding its value as a candidate therapeutic requires attention to how BTP2 may be tolerated in patients. While new SOCE channel inhibitors are in development for a range of human diseases, few have been FDA-approved due to a lack of specificity and potential toxicity. However, one known Orai channel blocker, CM4620 (Auxora), has reached phase II clinical trials for the treatment of acute pancreatitis (58) and coronavirus disease 2019 pneumonia (59). Additionally, a number of FDA-approved compounds, though not originally developed as Orai channel blockers, have since been shown to significantly reduce SOCE *in vitro* (60). These include leflunomide, teriflunomide, lansoprazole, tolvaptan, and roflumilast. Both leflunomide and teriflunomide demonstrated inhibitory activity against SOCE at their clinically relevant doses (60). Of note, these compounds were screened in part based on their structural similarity to BTP2. BTP2 has also been evaluated in preclinical mouse, rat, and guinea pig models of autoinflammatory disease after acute oral or intraperitoneal treatment and is well tolerated up to 30 mg/kg when administered perorally (54, 61–65). In addition to standard animal models, organoids are becoming an increasingly important model for preclinical compound validation. BTP2 treatment of *ex vivo* human intestinal epithelial organoids at 1 µM concentration did not significantly alter cell viability, cell differentiation, or barrier function (66). In addition, our work demonstrates that >80% human jejunal organoid viability is maintained with 30 µM BTP2 treatment.

Excitingly, our studies demonstrate that BTP2 has antiviral activity against GII.4 and GII.3 strains of HuNoV. To better contextualize its efficacy, we compared the antiviral activity of BTP2 to a positive control, 2-CMC, and determined its selective index. We used 2-CMC as the positive control because it is a nucleoside analog originally developed as a hepatitis C virus (HCV) antiviral and significantly inhibits MNV replication (67) and HuNoV in the Norwalk replicon system (68), B cells (69), and HIOs (38, 39, 70). Currently, 2-CMC is one of the most selective and potent anti-norovirus compounds identified to date; however, its prodrug, valopicitabine, failed phase II clinical trials in the treatment of HCV due to gastrointestinal toxicity (71). Thus, while 2-CMC is not a viable antiviral candidate, it represents a strict comparator for evaluating compound efficacy *in vitro*. Selective indices are an important pharmacologic metric used to account for both the potency of an antiviral therapeutic (EC_50_) and its related toxicity (CC_50_) (38). We found that the selective indices of BTP2 for GII.4 Sydney (P16) and GII.3 (P21) HuNoV are 4.63 and 6.36, respectively. While there is no strict, universal cutoff for therapeutic screens, selective indices greater than or equal to 10 have been used to indicate potential clinical utility. In context, 2-CMC has shown a selective index greater than 31 against GII.4 Sydney in HIOs (38). Thus, while BTP2 itself may not have optimal selective activity, the strong inhibition of TV replication and broad, albeit lesser, antiviral activity against HuNoV strains support the idea that BTP2 may be a promising starting pharmacophore that can be used to optimize or screen other candidate norovirus antiviral therapeutics.

Together, these studies define an important SOCE-independent role of the Orai channel blocker, BTP2, in inhibiting virus replication. In addition, our work demonstrates that BTP2 is a new, optimizable therapeutic for HuNoV infection. More broadly, we establish the utility of targeting norovirus structural proteins in antiviral development and present a platform whereby candidate antiviral compounds can be screened in surrogate virus systems, like TV, and validated with HuNoV infection in organoids. Together, by leveraging and expanding the tractability of surrogate virus systems to uncover the mechanistic underpinnings of candidate antivirals and capitalizing on the biological relevance of HIOs, we have developed an experimental framework that will facilitate the discovery of other novel antiviral therapies.

## MATERIALS AND METHODS

### Cell lines and viruses

MA104 African Green Monkey kidney epithelial cells were engineered to stably express the cytosolic calcium indicator, GCaMP6s, by lentivirus transduction as previously described (72). MA104G6s STIM1 knockout cells were cloned by limiting dilution and validated in an SOCE assay and via western blot (Fig. S1). Cells were incubated at 37°C in 5% CO_2_ and maintained in Dulbecco’s Modified Eagle Medium (DMEM) supplemented with 10% fetal bovine serum (FBS) and antibiotic-antimycotic (Gibco, 1× final concentration). TV was generated by serial passage in MA104 cells. All experiments were performed with a virus that has been passaged at least 19 times. TV stocks were generated by infecting MA104 cells at MOI 0.01 and harvesting 48 hpi (when ~95% CPE was observed). The virus was titrated by plaque assay on MA104 cells (see below). GII.4(P31) and GII.3(P21) HuNoV stocks were prepared as 10% stool filtrates as described previously (7).

### Human intestinal organoids

jHIOs were obtained from the Gastrointestinal Experimental Model Systems Core at the Texas Medical Center Digestive Diseases Center. HIOs suspended in Matrigel were cultured in “WRNE” culture medium containing Wnt3a, R-spondin-3, Noggin, EGF, and passaged every 7 days. HIOs were dispersed into a single cell suspension and plated as monolayers prior to differentiation for HuNoV infection studies. HIO differentiation was achieved by placing monolayers in Intesticult differentiation medium (STEMCELL Technologies) for 5 days before infection (6, 73).

### Compounds

BTP2 was purchased from Millipore Sigma (CAS 223499-30-7), Ro2959 from MedChemExpress (CAS 2309172-44-7), GSK-7975A from AOBIOUS (CAS 1253186-56-9), and Synta66 from Millipore Sigma (835904-51-3). Ro2959 was used at a final concentration of 5 µM, and BTP2, GSK, and Synta66 were used at a final concentration of 10 µM. In experiments where BTP2 was added to the plaque assay overlay, a final concentration of 5 µM was used to avoid cytotoxicity with >48 h of incubation. All compounds were dissolved in DMSO, which served as our vehicle control for all experiments. Thapsigargin was purchased from Thermo Scientific (CAS 67526-95-8) and used at a final concentration of 1 µM.

### SOCE assays

MA104G6s cells were seeded on µClear 96-well imaging plates (Greiner) at a density of 50,000 cells per well in 10% FBS DMEM maintenance medium. Once confluent, the cells were incubated with Orai channel inhibitors or DMSO for 20 min before imaging. After pre-treatment, cell monolayers were washed with calcium-free Hanks’ Balanced Salt Solution (HBSS) and equilibrated in the epifluorescence microscope live cell imaging chamber. Imaging was performed with a Nikon TiE inverted microscope with a SPECTRAX LED light source (Lumencor) using a 10X Plan Apo (NA 0.30) objective. Cells were imaged in a 2 s interval for 1–2 min to establish a baseline fluorescence before perfusion of 1 µM thapsigargin solution in calcium-free HBSS, again containing Orai channel inhibitors or DMSO. Following an additional 6–8 min of continuous imaging, the perfusion solution was then changed to calcium-containing HBSS (2 mM calcium) with inhibitor or control. The cells were imaged for an additional 4–5 min, after which the imaging run was completed. SOCE traces were generated by plotting the GCaMP6s fluorescence per field-of-view (FOV) over time. The maximum GCaMP6s delta fluorescence was calculated by subtracting the baseline normalized fluorescence of the FOV from the maximum fluorescence intensity following 2 mM calcium addition and plotting this relative to the DMSO control.

### Cytotoxicity assays

Cytotoxicity was measured in MA104 cells or J2 jHIOs using the Promega CytoTox 96 Non-Radioactive Cytotoxicity assay, according to the manufacturer’s instructions with minor modifications for HIOs (74). All experimental and control compounds were incubated with the monolayers at the indicated concentrations for 24 h. jHIOs were differentiated for 5 days prior to the addition of the compounds, and all jHIO supernatants and controls were diluted 1:10 prior to running the assay as previously described (74).

### Virus infections

MA104 monolayers were incubated in serum-free DMEM for 24 h prior to infection. TV inoculum was prepared at the indicated MOI in serum-free DMEM and absorbed on cell monolayers for 1 h at 37°C in 5% CO_2_. Following incubation, the virus inoculum was removed, and cell monolayers were washed with PBS before adding the maintenance medium. HuNoV infections were performed on differentiated HIO monolayers in a 96-well plate pre-treated for 1 h with vehicle control, BTP2, or 50 µM 2-CMC. Cells were infected with 100 TCID_50_ of GII.3 or GII.4 HuNoV diluted in CMGF (Advanced DMEM F12, 1× Glutamax, 10 mM HEPES, 100 U/mL penicillin/streptomycin). After a 1 h inoculum incubation, cells were washed with CMGF and placed in Intesticult (Stem Cell Technologies) differentiation medium containing BTP2 at the indicated concentrations or 50 µM 2-CMC. At 24 hpi, cell lysates and supernatants were harvested for lactate dehydrogenase (LDH) cytotoxicity assay or quantitative reverse transcription PCR (RT-qPCR).

### Virus yield assays

MA104 cells were seeded in 24-well tissue culture-treated plates at a density of 100,000 cells per well and grown to confluency. Cells were infected with TV at an MOI of 1 or 10 as described above. Following inoculum incubation, cells were placed in medium containing Orai channel inhibitors or DMSO vehicle control. At 24 hpi, cells were harvested by three freeze-thaw cycles, and virus was quantitated by plaque assay. For plaque assays, MA104 cells were seeded in a 6-well tissue culture plate at a density of 500,000 cells per well and grown to confluency. Cells were inoculated with 10-fold serial dilutions of infected lysates as described above. Following infection, cell monolayers were overlaid with a 1.2% microcrystalline cellulose solution, made by combining equal parts 2.4% microcrystalline cellulose (Avicel) with 2× DMEM, and supplementing with 0.1 mg/mL DEAE dextran. Plaque assays were harvested 72 hpi, and monolayers were stained with crystal violet to visualize plaques.

### Virus growth curves

MA104 cells were seeded in 24-well tissue culture-treated plates at a density of 100,000 cells per well and grown to confluency. Cells were infected with TV (parental or serial plaque isolated) at an MOI of 1 as described above. Following inoculum incubation, cells were placed in medium containing Orai channel inhibitors or DMSO vehicle control at the indicated time points, 1, 6, 12, and 24 h after infection. Cells were harvested by three freeze-thaw cycles, and the virus was quantitated by plaque assay.

### Western blot

MA104 cells were mock inoculated or infected with BTP2-susceptible TV (susceptible clone 2) at an MOI of 1 as described above and treated with either DMSO or 10 µM BTP2 at 1, 6, or 10 hpi. Cell lysates were harvested at 24 hpi in radioimmunoprecipitation assay (RIPA) buffer (50 mM Tris base, 150 mM NaCl, 1% NP-40, 0.5% sodium deoxycholate, and 0.1% sodium dodecyl sulfate). Samples were homogenized using a biopolymer column (QiaShredder, Qiagen), combined with SDS-PAGE sample buffer, and heated at 100°C for 10 min prior to loading. Proteins were separated on 4%–20% Tris-glycine SDS-PAGE gels (BioRad) and transferred onto a nitrocellulose membrane. Blocking was performed with 10% non-fat dry milk in PBS. Primary antibodies (Rabbit αTV VP1, synthesized by ABClonal; mouse αGAPDH, Southern Biosciences; FLAG) were diluted in 0.5% blocking solution, used at a concentration of 1:2,000, and incubated overnight at room temperature. Secondary antibodies (Goat αRabbit-AP, Goat αMouse-AP) were also diluted in 0.5% blocking solution, used at a concentration of 1:2,000, and incubated for 2 h at room temperature. Membranes were washed with 0.5% blocking solution and developed with an alkaline phosphatase detection solution containing 50 mM Tris, 3 mM MgCl_2_, 0.1 mg/mL p-nitro blue tetrazolium chloride, and 0.05 mg/mL 5-bromo-4-chloro-3-indolyl phosphate. Blots were imaged using a document scanner (Canon), and band intensity was quantified by converting blots to 8-bit grayscale images in ImageJ, inverting the pixel intensity, performing uniform background subtraction, and taking the ratio of the sum intensity of each band to that of the respective GAPDH loading control. Quantitation is from at least three blots run with lysate from at least three independent infections.

### TV BTP2 plaque assays

MA104 cells were seeded in 6-well plates at a density of 500,000 cells/well and grown to confluency. Cells were inoculated with 10-fold serial dilutions of virus stock (3 × 10^7^ PFU/mL to 3 × 10^8^ PFU/mL, depending on MA104 passage number) as described above. A 3 mL solution of 1.2% microcrystalline cellulose overlay containing 5 µM BTP2 or an equivalent volume of DMSO was added to each of the wells. Plaque assays were harvested 72 hpi, and monolayers were stained with 1× crystal violet to visualize plaques. Plaque size was quantitated by measuring the diameter of well-isolated plaques.

### Solid overlay plaque assays and plaque picking

TV solid overlay plaque assays were performed as described above, with the modification that a 1.2% agarose and neutral red overlay was used in place of the microcrystalline cellulose overlay. The first SeaKem agarose overlay was made by combining equal parts 1.2% SeaKem agarose (SeaKem LE agarose, Lonza) solution and 2× DMEM medium, supplemented with DEAE dextran and either 5 µM BTP2 or DMSO. Forty-eight hours after the first overlay, a second overlay containing equal parts 1.2% SeaKem agarose solution and 2× DMEM medium, supplemented with 0.05% neutral red, was added to each of the wells. Twenty-four hours after the second overlay was added, plaques were picked by harvesting the overlay plug and adding it to 1 mL of serum-free DMEM. Plaque picks were then used to inoculate MA104 cells to generate stocks in the presence of DMSO or 10 µM BTP2 for downstream yield, plaque, and CPE inhibition assays.

### Cytopathic effect virus inhibition assay

The CPE inhibition assay was performed as previously described (75). Briefly, MA104 cells were grown to confluency in 96-well tissue culture plates. Once confluent, cell monolayers were inoculated with TV at an MOI of 3 or 10, as described above. Infected monolayers were treated with DMSO or BTP2 at the indicated concentrations and incubated for 24 h, after which maintenance medium was removed and the cells were washed with PBS. Monolayers were then stained with 1× crystal violet for 10 min with rocking. The unbound stain was removed with PBS wash, and monolayers were allowed to dry at room temperature. Bound crystal violet stain was eluted with 33% acetic acid (100 µL per well), which was incubated for 10 min at room temperature, with rocking. From each well, 100 uL of crystal violet elution was transferred to a 96-well plate, and absorbance was measured at 630 nm (BioTek ELx808 spectrophotometer). The inhibition rate (%) was calculated with the following equation: inhibition rate = [(OD_inhibitor-treated infected cells_ − OD_DMSO-treated infected cells_)/(OD_DMSO-treated uninfected cells_ − OD_DMSO-treated infected cells_)] × 100.

### Virus drug passaging

Passage 1 TV was generated by infecting MA104 cell monolayers with TV at an MOI of 1 as described above. After inoculation, monolayers were incubated in serum-free medium containing DMSO vehicle control or 10 µM BTP2. Infected lysate was harvested by three freeze-thaw cycles at 24 hpi. Passage 2–4 TV was generated by infecting MA104 monolayers in a 24-well plate with 200 uL of lysate from the previous passage. The lysate was allowed to absorb for 3 h at 37°C in 5% CO_2_. After the inoculation period, the lysate was removed, monolayers were washed with phosphate-buffered saline (PBS), and medium was replaced with serum-free DMEM containing DMSO control or 10 µM BTP2. Infected lysate was harvested at ~32 hpi by three freeze-thaw cycles.

### TV VP2 transduction and co-infection

AAV vectors expressing mCherry and 3X FLAG-tagged TV VP2 or mScarlet alone were commercially synthesized and packaged (VectorBuilder). For transduction, MA104 cells were seeded in a 24-well plate at a density of 150,000 cells per well. One day post-seeding, cell monolayers were transduced with susceptible or resistant VP2 (clone 1) at an MOI of 25,000 genome copies per cell or mScarlet at an MOI of 1,000 genome copies per well. Forty-eight hours post-transduction (hpt), cells were imaged to assess transduction efficiency and subsequently harvested in RIPA buffer or infected with TV (susceptible clone 1) at an MOI of 1. Infected cells were treated with either DMSO or 10 µM BTP2 1 hpi. After 24 h, infected cell lysates were harvested and titrated via plaque assay, as described above.

### TV sequencing

RNA was isolated from MA104 cells infected with the serial plaque isolated virus at MOI 3 in the presence of either DMSO or BTP2 using a viral RNA kit (Zymogen), according to the manufacturer’s instructions. RNA yield and quality were assessed on a spectrophotometer (Nanodrop, Thermo Scientific). cDNA synthesis and genome amplification were done in two segments, an ORF1 and an ORF2,3 segment, following the manufacturer's protocol (One-Step RT-PCR kit, Takara). PCR product size was verified by gel electrophoresis. Primers used are listed in Table 1. PCR products were column-purified (Promega Wizard PCR Cleanup Kit) and sequenced by Sanger sequencing (Azenta). Sequencing primers are listed in Table 2. Sequencing contigs were assembled and aligned using SnapGene (ver. 7.2.0) software.

**TABLE 1:** Primers for TV one-step RT-PCR

Primer	Sequence
ORF1	
Forward	GTGACTAGAGCTATGGATACGTC-3
Reverse	GTCTATGACAATTGTGATCAATCACAAG-3
ORF2,3	
Forward	CGTGGTTCGGTGAGC-3
Reverse	GCTTAGGGTCTCACTCCGG-3

**TABLE 2:** Primers used for Sanger sequencing

Primer	Sequence
Forward	GTGACTAGAGCTATGGATACGTC-3
Forward	GTCAAAGATGTCAATTGGGCAAAG-3
Forward	GATCCCTCTGCACCGC-3
Forward	GCAACGGACCAAAGCCATAC-3
Forward	CTTCCTCAAAGCAAATGCTGTC-3
Forward	GTTAAATCCCAAACCAAACTTGAGG-3
Forward	GGAAGAGTGAACCAAGCAG-3
Forward	GATTGGTGTCAAAACACTCTTTG-3
Forward	CGTGGTTCGGTGAGC-3
Forward	GCAGGCAATGCCTTCTC-3
Forward	GCACAGCAGAAGCCG-3
Forward	CTGATGTCACCTGACCTTATGTG-3
Forward	CTGATGCTGCTCTTATGTCTAAG-3
Reverse	GCTTAGGGTCTCACTCCGG-3

### Quantitating TV genome equivalents through RT-qPCR

MA104 cells were seeded in a 24-well plate and grown to confluency. Cells were infected with TV (susceptible clone 1) at MOI 1 and treated with BTP2 1 h before inoculation, during inoculation, or at 1, 6, or 10 h post-inoculation. At 24 hpi, cells were harvested, and RNA was isolated using column-based extraction (Zymogen viral RNA kit) per the manufacturer’s instructions. RNA yield and quality were assessed on a spectrophotometer (Nanodrop, Thermo Scientific). cDNA was synthesized from 800 ng of RNA (SensiFast cDNA synthesis kit, Bioline USA Inc.). qPCR was performed by dye incorporation (SYBR green, Invitrogen) and fluorescence quantification (QuantStudio, Applied Biosystems). Each sample was run in technical duplicate. Viral RNA copy number was calculated based on the CT value of the sample compared to a standard curve of total *in vitro* transcribed RNA with known copy numbers. Primer pairs for detection of TV at the 5′ end of the genome were forward: 5- GTCAAAGATGTCAATTGGGCAAAG-3 and reverse: 5-CCCAAGGCACCCAAAACC-3.

### Quantitating HuNoV genome equivalents through RT-qPCR

RNA was extracted from infected HIO monolayers using the MagMAX-96 viral RNA isolation kit on the Kingfisher Flex machine (Thermo Scientific). cDNA synthesis and PCR amplification were performed using the qScript XLT One-Step RT-qPCR ToughMix kit (Quantabio) with the following primer pair and probe sets: COG2R/QNIF2d/QNIFS. Viral RNA copy number was calculated based on the CT value of the sample compared to a standard curve based on recombinant GII.4 Houston virus RNA transcripts (7).

### Fluorescence resonance energy transfer (FRET) protease assays

Tulane protease and GII.4 Sydney protease were expressed in *E. coli* with an N-terminal 6xHis-TELSAM fusion tag with an HRV3C protease cleavage site and purified by Ni-NTA chromatography, tag cleavage and removal, and size exclusion chromatography. A total of 2.5× solutions of protease (Tulane: 5 µM, GII.4 Sydney: 2.5 µM) and 2× solutions of FRET substrate [Glu(EDANS)-GDYELQGPEDLA-Lys(Dabcyl), 40 µM] were prepared by dilution of more concentrated stocks with assay buffer (10 mM HEPES, 30% glycerol, 10 mM DTT, 0.1% CHAPS, pH 8.0). A total of 10× stocks of BTP2 of various concentrations were diluted from a 10 mM BTP2 stock with DMSO. Assays were run with 100 uL reaction volumes in 96-well black NBS plates (Corning 3991) at 37°C and read with the FlexStation 3 (Molecular Devices) with 90 s measurement intervals (excitation 340 nm, emission 490 nm, and filter 475 nm).

### Cell-free polymerase assay

GII.4 RdRp activity was measured using a real-time fluorescence-based assay, which uses SYTO9, a fluorescence dye that specifically binds dsRNA but not ssRNA template molecules. Reactions were performed in individual wells of black 96-well flat-bottom plates (Costar). The standard reaction contained GII.4 RdRp (1 µM), 20 mM Tris-HCl pH 7.5, 5 mM MgCl_2_, 2.5 mM MnCl_2_, 40 µg/mL polyC, 5 U RNAseOUT (Invitrogen), and 0.25 µM SYTO9 (Sigma-Aldrich). The reaction was initiated by the addition of 300 µM GTP, and the fluorescence was recorded every 5 min over 180 min at 37°C using a plate reader FlexStation3 (Molecular Devices). To investigate the effect of BPT2 on the polymerization activity of RdRp, 1 µM RdRp was incubated with 100 µM BPT2 at room temperature for 1 h, and the RdRp activity was measured using the above-mentioned protocol.

### Tulane virus reverse genetics

cDNA plasmids encoding the TV genome with the BTP2-susceptible or resistant-associated VP1 and/or VP2 sequences downstream of a CBh RNA PolII and K1E phage promoter were synthesized commercially (Vector Builder). MA104 cells were transfected with 7.5 ug of the TV genomic DNA plasmid and 2.5 ug of C3P3, a T7 polymerase-African swine fever virus capping enzyme fusion protein (76). Transfections were performed with the TransIT-LT1 transfection reagent at a concentration of 2 µL per 1 µg of DNA, per the manufacturer’s instructions. CPE was observed ~72 hpt, upon which cells were harvested by three freeze/thaw cycles. The rescued virus was passaged twice in MA104 cells, titrated by plaque assay, and utilized for BTP2 yield and dose range studies as described above. Viral sequences were confirmed by Sanger sequencing (Azenta).

## Data Availability

The data that support the findings of this study are available from the corresponding author, J.M.H., upon reasonable request.

## SUPPLEMENTAL MATERIAL

Supplemental figures jvi.00444-25-s0001.docx Figures S1 to S6.

ASM does not own the copyrights to Supplemental Material that may be linked to, or accessed through, an article. The authors have granted ASM a non-exclusive, world-wide license to publish the Supplemental Material files. Please contact the corresponding author directly for reuse.
