# Supplementary material for: BTP2 restricts Tulane virus and human norovirus replication independent of store-operated calcium entry
Source: J Virol. 2025 May 29;99(6):e00444-25. doi: 10.1128/jvi.00444-25 (PMC12172428; doi:10.1128/jvi.00444-25)
Supplement: Supplemental figures — Figures S1 to S6. [file jvi.00444-25-s0001.docx]

**Supplemental Figures**


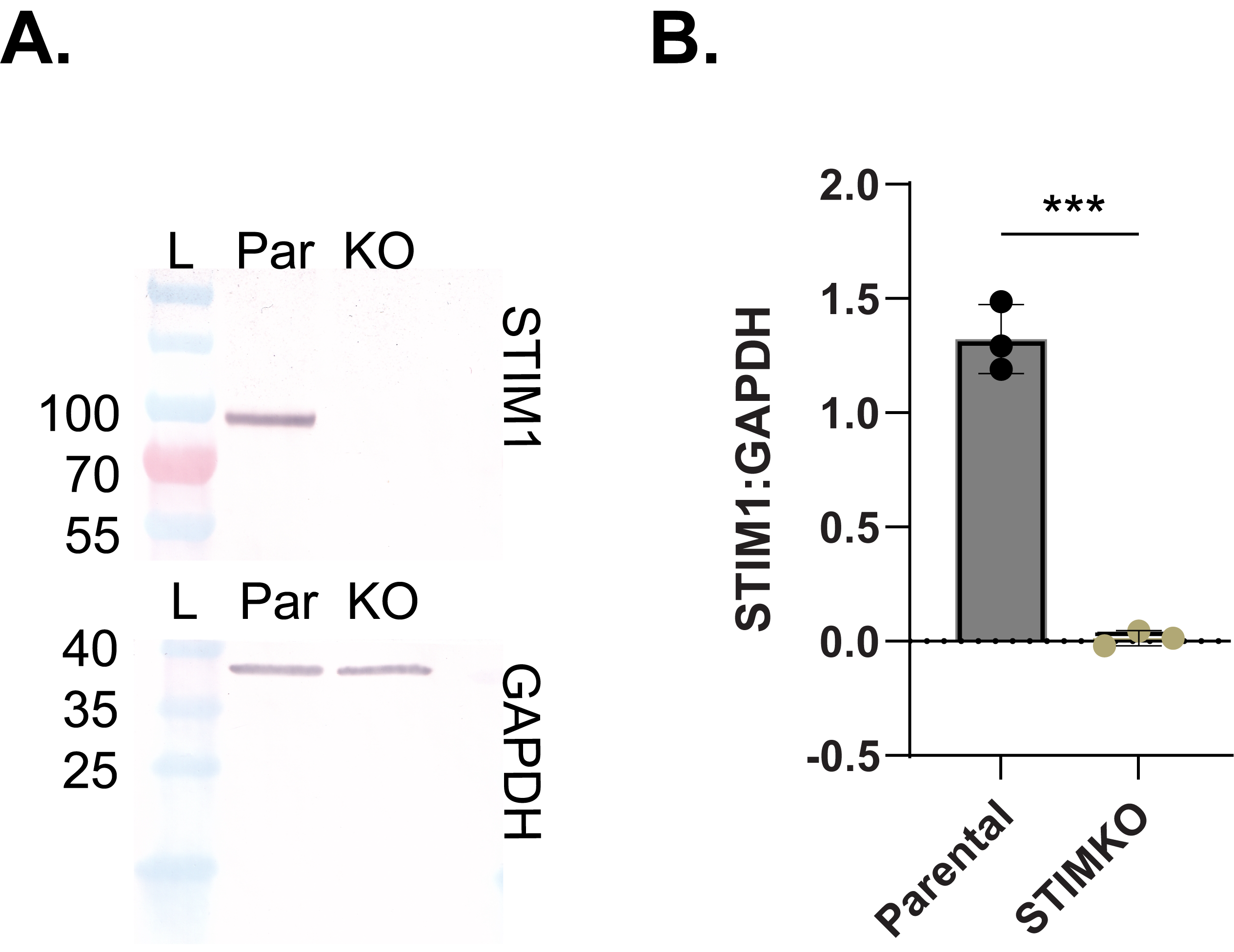


**Supplemental Figure 1: MA104G6s STIM1KO cells have undetectable STIM1 protein expression. A**) STIM1 protein expression (top) detected by Western in MA104G6s parental (Par) or STIM1 knockout (KO) cells. GAPDH (bottom) served as the loading control. **B**) Quantitation of band intensity plotted relative to GAPDH. Each point represents an independent biological replicate. **p<0.01 by unpaired T test.


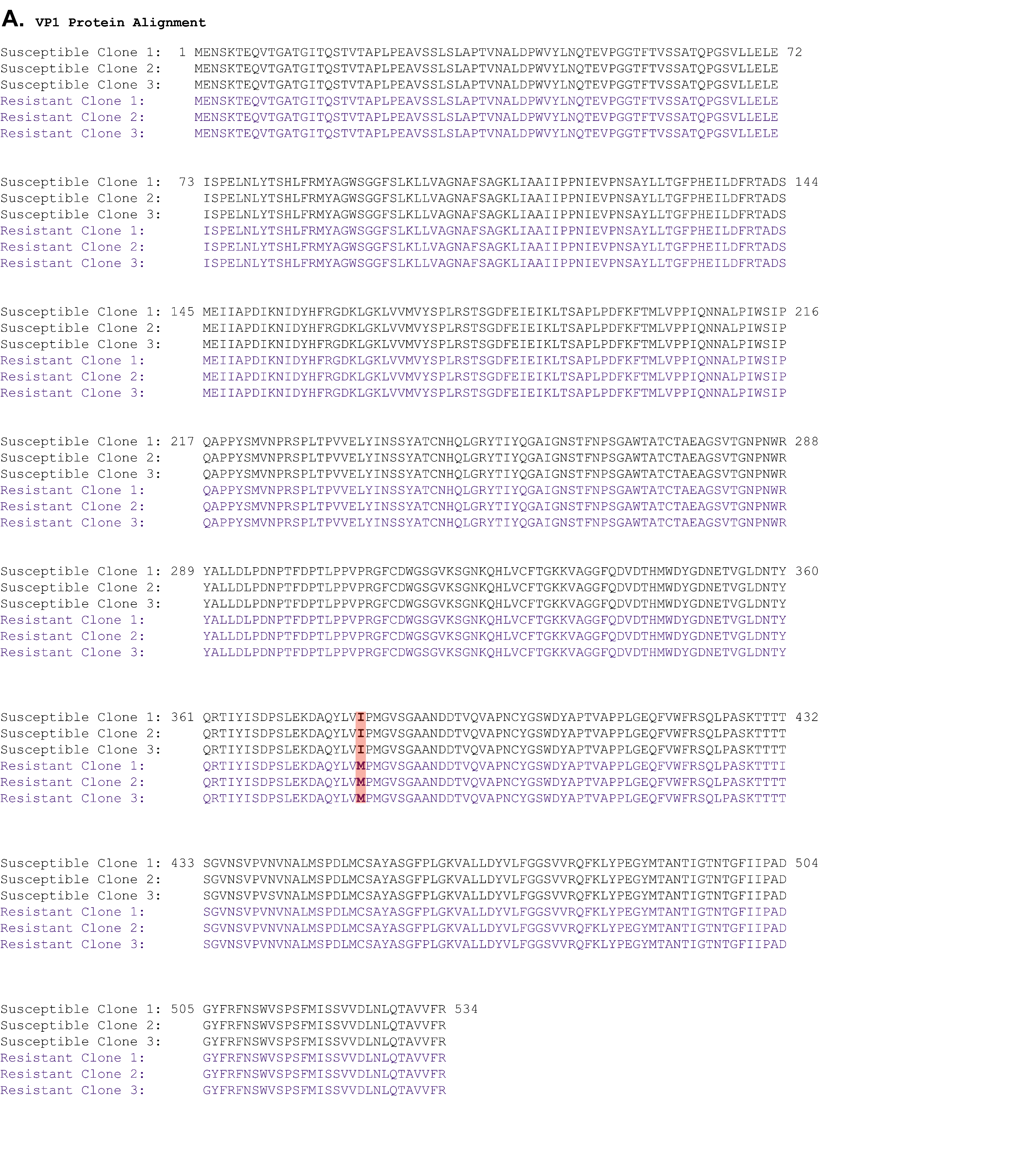


**Supplemental Figure 2: VP1 amino acid alignment.**

**A**) VP1 amino acid alignment between 3 BTP2 susceptible and 3 BTP2 resistant variants. Conserved amino acid differences are highlighted in red.


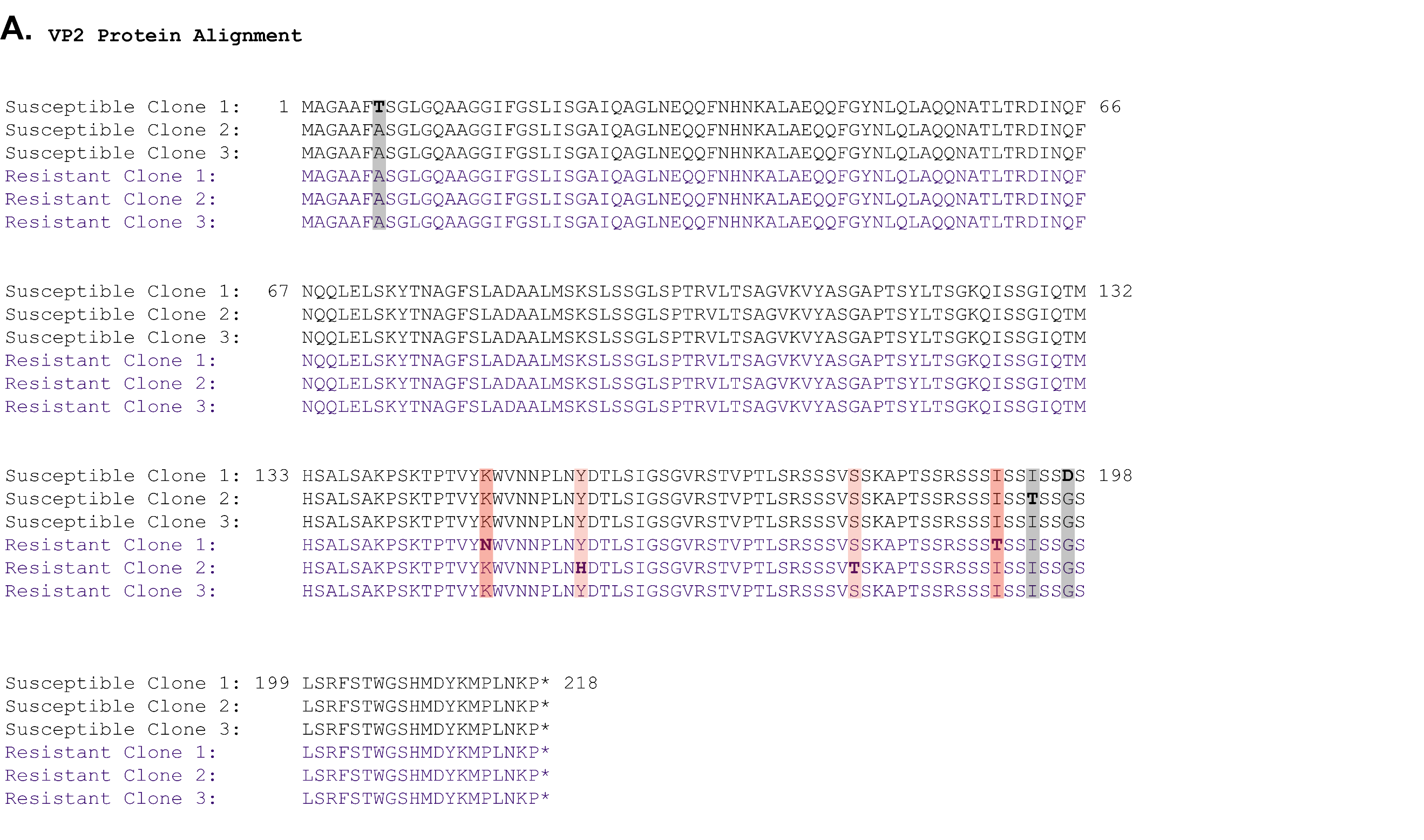


**Supplemental Figure 3: VP2 amino acid alignment.**

**A**) VP2 amino acid alignment between 3 BTP2 susceptible and 3 BTP2 resistant variants. Amino acids that differ from the consensus sequence in the resistant (red) or susceptible (grey) clones are highlighted.


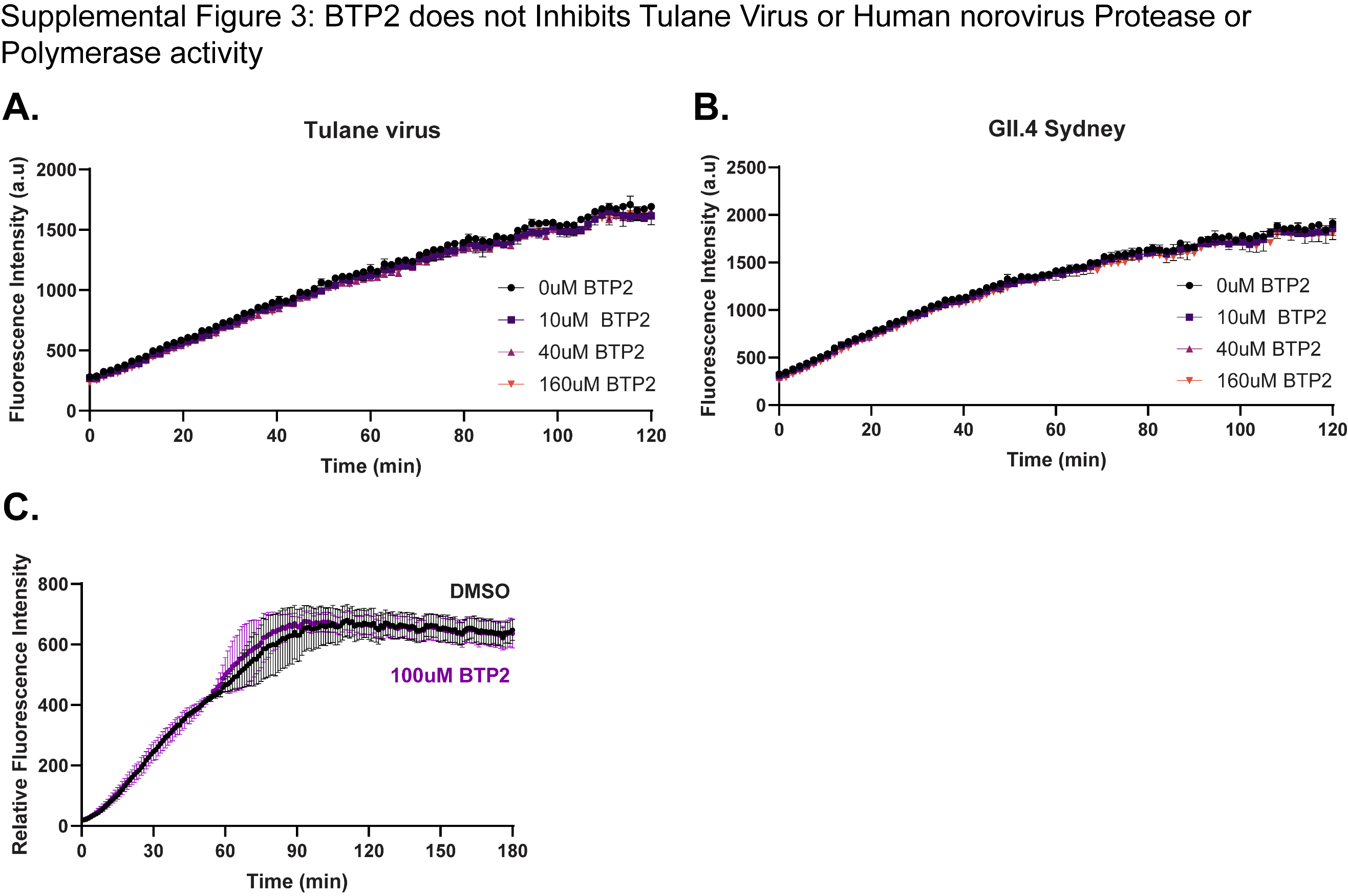


**Supplemental Figure 4:** **BTP2 does not inhibit TV or HuNoV Protease or Polymerase activity.** **A-B**) Cell free FRET assay measuring TV (A) or GII.4 Sydney HuNoV (B) protease activity in the absence (black) or presence of BTP2 at the 10µM (purple), 40µM (pink), or 160µM (orange) concentration. **C**) GII.4 RDRP activity in the presence of DMSO (black) or 100µM BTP2 (purple).


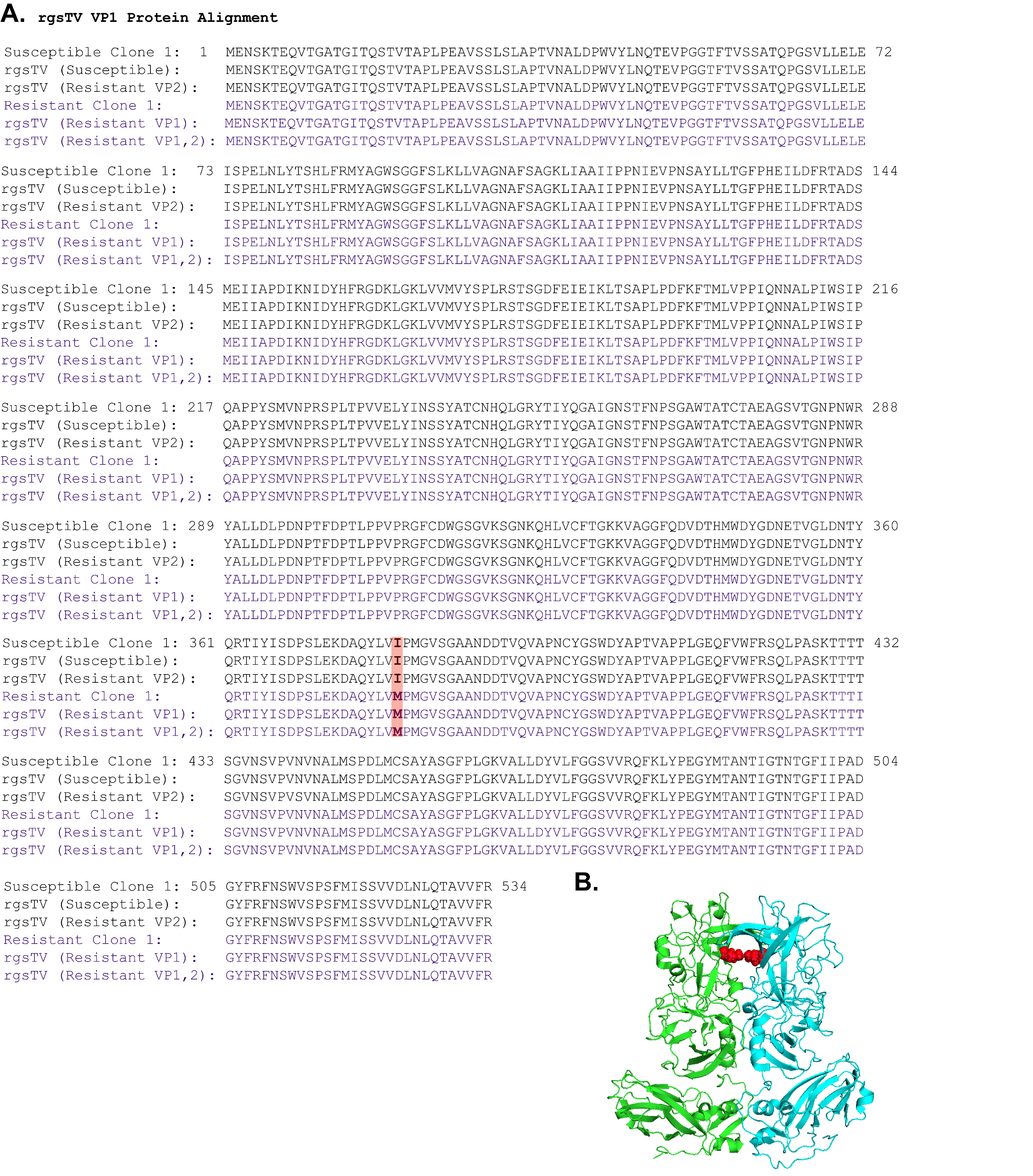


**Supplemental Figure 5: Reverse genetics TV VP1 amino acid alignment.**

**A**) VP1 amino acid alignment of the clone 1 BTP2 susceptible and clone 1 BTP2 resistant TV sequences with the reverse genetics Tulane viruses. **B**) Mapping of the isoleucine at position 380 (red) on the VP1 dimer.


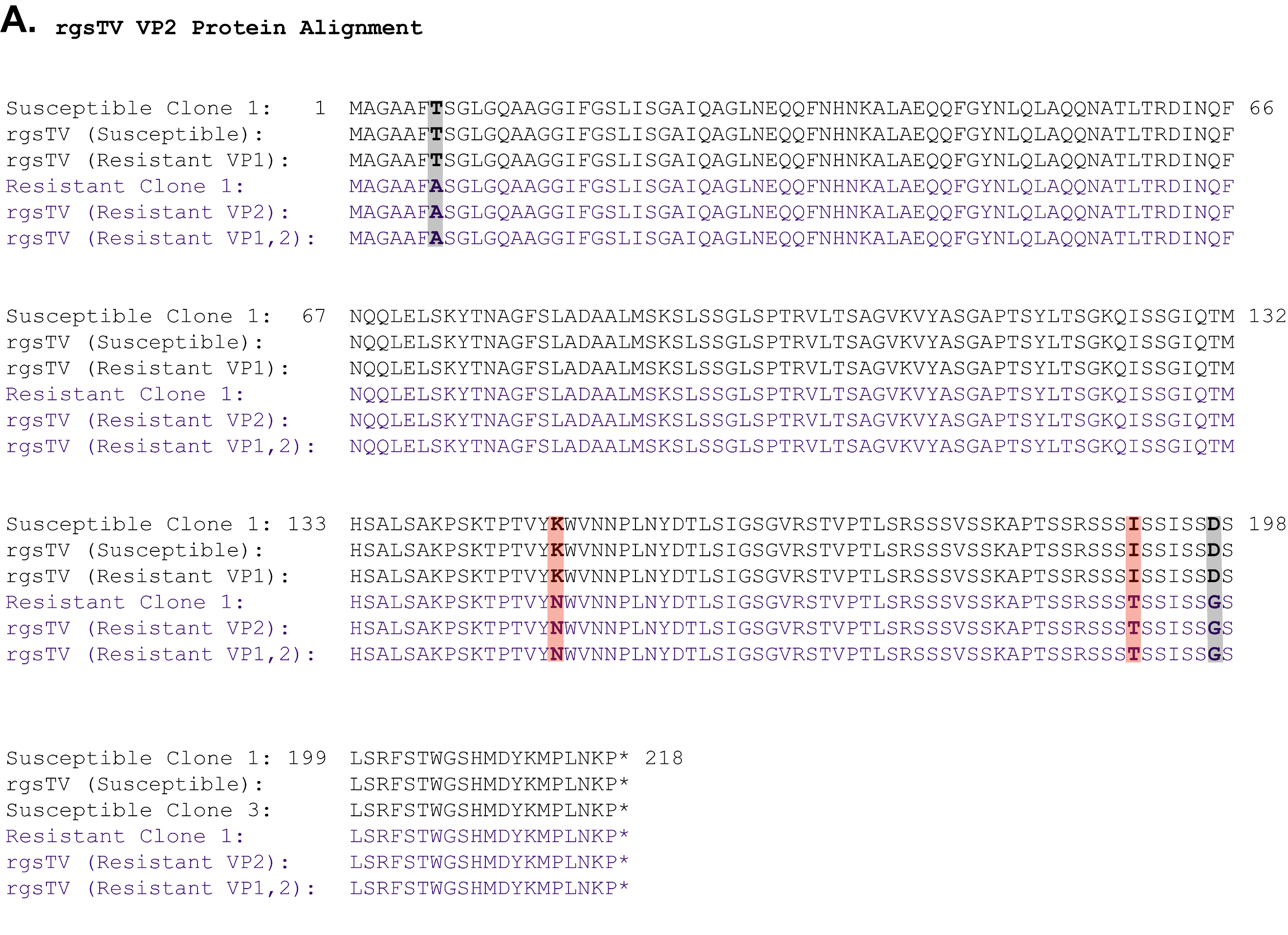


**Supplemental Figure 6: Reverse genetics TV VP2 amino acid alignment.**

**A**) VP2 amino acid alignment of the clone 1 BTP2 susceptible and clone 1 BTP2 resistant TV sequences with the reverse genetics Tulane viruses.
